# Structural basis for binding of the renal carcinoma target hypoxia‐inducible factor 2α to prolyl hydroxylase domain 2

**DOI:** 10.1002/prot.26541

**Published:** 2023-07-14

**Authors:** William D. Figg, Giorgia Fiorini, Rasheduzzaman Chowdhury, Yu Nakashima, Anthony Tumber, Michael A. McDonough, Christopher J. Schofield

**Affiliations:** ^1^ Chemistry Research Laboratory, Department of Chemistry and the Ineos Oxford Institute for Antimicrobial Research, University of Oxford Oxford UK; ^2^ Institute of Natural Medicine, University of Toyama Toyama Japan

**Keywords:** Belzutifan, clear cell renal cell carcinoma, erythropoiesis, hypoxia‐inducible factor isoform 2‐alpha (HIF2α or EPAS1), prolyl hydroxylase domain (PHD or EGLN), *Trichoplax adhaerens* and *Pseudomonas putida* prolyl hydroxylase domain (*Ta*PHD and PPHD), α‐ketoglutarate/2‐oxoglutarate oxygenase

## Abstract

The hypoxia‐inducible factor (HIF) prolyl‐hydroxylases (human PHD1‐3) catalyze prolyl hydroxylation in oxygen‐dependent degradation (ODD) domains of HIFα isoforms, modifications that signal for HIFα proteasomal degradation in an oxygen‐dependent manner. PHD inhibitors are used for treatment of anemia in kidney disease. Increased erythropoietin (EPO) in patients with familial/idiopathic erythrocytosis and pulmonary hypertension is associated with mutations in *EGLN1* (PHD2) and *EPAS1* (HIF2α); a drug inhibiting HIF2α activity is used for clear cell renal cell carcinoma (ccRCC) treatment. We report crystal structures of PHD2 complexed with the C‐terminal HIF2α‐ODD in the presence of its 2‐oxoglutarate cosubstrate or N‐oxalylglycine inhibitor. Combined with the reported PHD2.HIFα‐ODD structures and biochemical studies, the results inform on the different PHD.HIFα‐ODD binding modes and the potential effects of clinically observed mutations in HIFα and PHD2 genes. They may help enable new therapeutic avenues, including PHD isoform‐selective inhibitors and sequestration of HIF2α by the PHDs for ccRCC treatment.

## 1 INTRODUCTION

**FIGURE 1:**
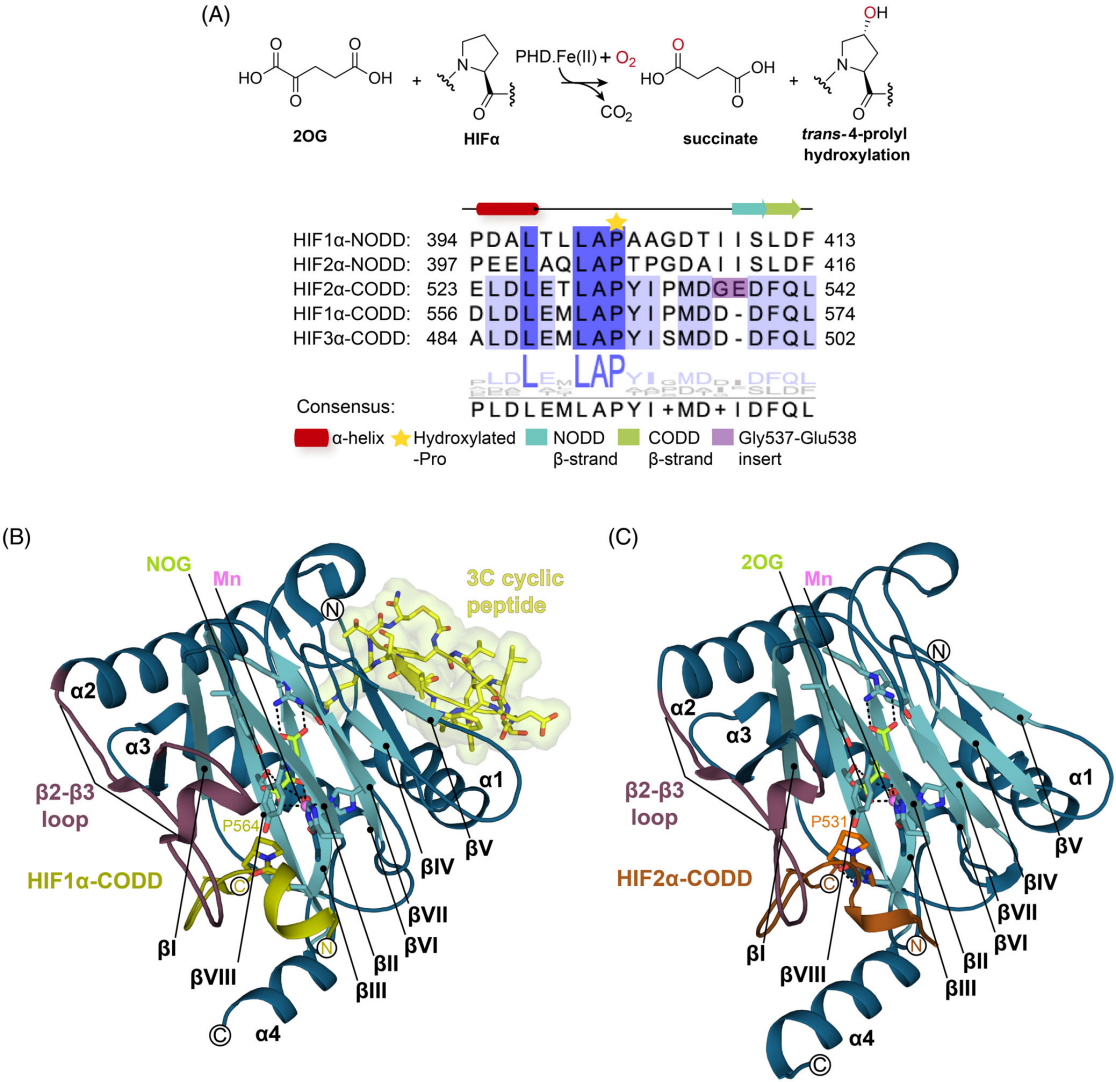
Overview of HIFα prolyl hydroxylase catalysis and view of the conserved double‐stranded β‐helix fold of the 2OG oxygenases. (A) PHD1‐3 catalyze 2OG‐dependent *trans*‐4‐prolyl hydroxylation of HIFα isoforms. Sequence alignment of the five human HIFα N‐/C‐terminal oxygen‐degradation domains. The secondary structure (α‐helix: red and β‐strand: orange‐NODD/green‐CODD) assignments are as observed in crystal structures of HIF1α_394–413_‐NODD and HIF1α_556–574_‐CODD in complex with the PHD2 catalytic domain (PDB: 5L9V and 5L9B). The hydroxylated proline is marked with a yellow star. (B, C) The distorted double‐stranded β‐helix core fold (teal βI‐βVIIΙ refer to the eight DSBH strands), the β2–β3 finger loop (red cartoon), and the N‐/C‐terminal extensions of the DSBH (N: α1–α3 and C: α4 helices‐blue) are labeled. (B) The PHD2_181‐407_.Mn(II).NOG.HIF1α‐CODD.3C (6YW3) complex is depicted as a cartoon showing the N‐terminal binding site of the co‐crystallized 3C cyclic peptide (yellow sticks with Connolly surface). (C) A view of the DSBH of the PHD2_181–407_.Mn(II).2OG.HIF2α‐CODD (7Q5X) complex, the main focus of this work. Key active site residues (teal), target proline (orange), and NOG (lemon) are represented as sticks. The HIF1α‐CODD (olive) and HIF2α‐CODD (orange) substrates are displayed as cartoons. Waters (red) and Mn (violet) are shown as spheres. CODD, C‐terminal oxygen‐dependent degradation; HIF, hypoxia‐inducible factor; NODD, N‐terminal oxygen‐dependent degradation.

In humans and other animals, the hypoxia‐inducible factor (HIF) transcription factors play key roles in responses to limiting O_2_ availability by promoting context‐dependent expression of genes working to alleviate the effects of hypoxia. HIF is an α,β‐heterodimeric protein; the levels of HIFβ, also known as the aryl hydrocarbon receptor nuclear translocator protein, are not regulated directly by O_2_ concentrations.^1^ By contrast, as a consequence of catalysis by the HIF prolyl hydroxylase domain enzymes (human PHD1‐3), HIFα levels are strongly regulated by O_2_ availability.^2^, ^3^ PHD1‐3 catalyze *trans‐*4‐prolyl hydroxylation of the N‐ and C‐terminal oxygen‐dependent degradation (NODD and CODD, respectively) domains in HIF1‐3α isoforms (note, HIF3α only contains a CODD) (Figure 1A). Such prolyl hydroxylation promotes binding of HIF1‐3α to the von Hippel–Lindau protein (pVHL) ubiquitin ligase complex, so signaling for proteasomal mediated hydrolysis of HIFα isoforms (Figure 1A).^4^, ^5^, ^6^, ^7^ A second HIFα hydroxylase, factor‐inhibiting HIF (FIH) catalyzes the C3 hydroxylation of an asparagine‐residue in the C‐terminal transcriptional activation domains of HIF1α and HIF2α (but not HIF3α), a modification that reduces the interaction of HIF with histone acetyltransferases (CREB binding protein and p300).^8^ The FIH catalyzed modification inhibits HIF‐mediated transcription in an incompletely understood context‐dependent manner (Figure S1).^8^, ^9^, ^10^, ^11^

In hypoxia, PHD1‐3 activity decreases and HIF1‐3α levels rise (Figure S1).^2^, ^3^ HIF1‐3α translocate to the nucleus and dimerize with HIFβ to form transcriptionally active α,β‐HIF heterodimers (Figure S1).^2^, ^3^, ^12^ These bind to hypoxia response elements (HREs) associated with HIF target genes and consequently upregulate transcription of HIF controlled genes including those encoding for erythropoietin (*EPO*) and vascular endothelial growth factor (*VEGF*) (Figure S1).^1^, ^2^, ^3^, ^13^

The PHDs and FIH are both Fe(II) and 2‐oxoglutarate (2OG)‐dependent oxygenases that couple hydroxylation with the conversion of 2OG to succinate and CO_2_ (Figures 1A and S1).^3^, ^8^, ^11^, ^14^, ^15^ Other human 2OG oxygenases have roles in the regulation of gene expression (e.g., the JmjC histone demethylases), and in other important cellular processes, including metabolism and collagen biosynthesis.^14^, ^16^, ^17^, ^18^ The biochemical properties of the PHDs are apparently suited to their roles as hypoxia/“O_2_‐sensors”; thus, they have unusually high *K*_m_ values for O_2_ and react relatively slowly with O_2_, compared to FIH and most other 2OG oxygenases.^19^, ^20^, ^21^, ^22^ PHD2 also forms a relatively stable complex with Fe and 2OG, even after exposure to O_2_. Collectively, these observations suggest that the biochemical properties of the PHDs may be focused to sense O_2_ availability.^19^, ^20^, ^21^, ^22^, ^23^ The PHDs are more sensitive than FIH to limiting O_2_ levels^24^ and HIFα‐NODD hydroxylation is reported to be more sensitive than CODD hydroxylation to O_2_ levels.^24^, ^25^ The PHDs also show different selectivity toward the various HIFα‐ODDs, with PHD3 being reported to be particularly selective for the HIF1α‐ and HIF2α‐CODD domains (Figures 1A and S1).^12^, ^22^, ^26^

Crystal structures of PHD2.HIFα‐ODD complexes and kinetic studies have revealed the importance of a conformationally mobile loop (the β2–β3 loop) that links β2 and β3 of the catalytic domain of PHD2 and which is involved in HIFα‐ODD binding and selectivity; in the PHD2.substrate complexes, the β2–β3 loop folds to isolate the HIFα‐ODD substrate proline at the active site.^11^, ^27^, ^28^, ^29^, ^30^ Overall, these observations support the proposal that β2–β3 loop dynamics are important both in catalysis and determining PHD/HIFα‐ODD substrate selectivity, though the precise molecular details are undefined (Figure 1B,C).^27^, ^29^, ^30^

*VHL* gene mutation is common in clear cell renal cell carcinoma (ccRCC) patients causing upregulation of HIFα isoforms, so increasing the expression of the HIF2α target *VEGF*, in a manner apparently promoting tumorigenesis and cancer progression.^31^, ^32^ Belzutifan (MK‐6482 or PT‐2977) inhibits HIF2α‐mediated expression and is used for ccRCC treatment (Figure S1).^33^ Mutations in *EPAS1* (encoding for HIF2α), *EGLN1* (encoding for PHD2), are also linked to disease, including familial/idiopathic erythrocytosis and ccRCC (Figure S2A).^34^ Thus, structural information of how the PHDs bind HIFα‐ODDs, and in particular HIF2α, may inform on the clinically observed pathologies of the mutant *EPAS1*‐related diseases.

Although structures of HIF1α‐CODD and NODD in complex with the catalytic domain of PHD2 are available, analogous structures with HIF2α‐CODD have not been reported.^11^, ^27^, ^28^ The PHD.HIF2α‐CODD complexes are of particular interest because of the disease relevance of HIF2α and because of differences with HIF1α/2α‐NODD and HIF1α/3α‐CODD. In particular, HIF2α contains an ‘additional’ (Gly) residue on the C‐terminal side of the hydroxylated proline, compared to HIF1α/3α‐CODD and HIF1‐2α‐NODD (Figure 1A). In the respective position in HIF1/2α‐NODD, the polar Gly537/Glu538 unit in HIF2α is substituted by two non‐polar Ile‐residues (Figure 1A).

**FIGURE 2:**
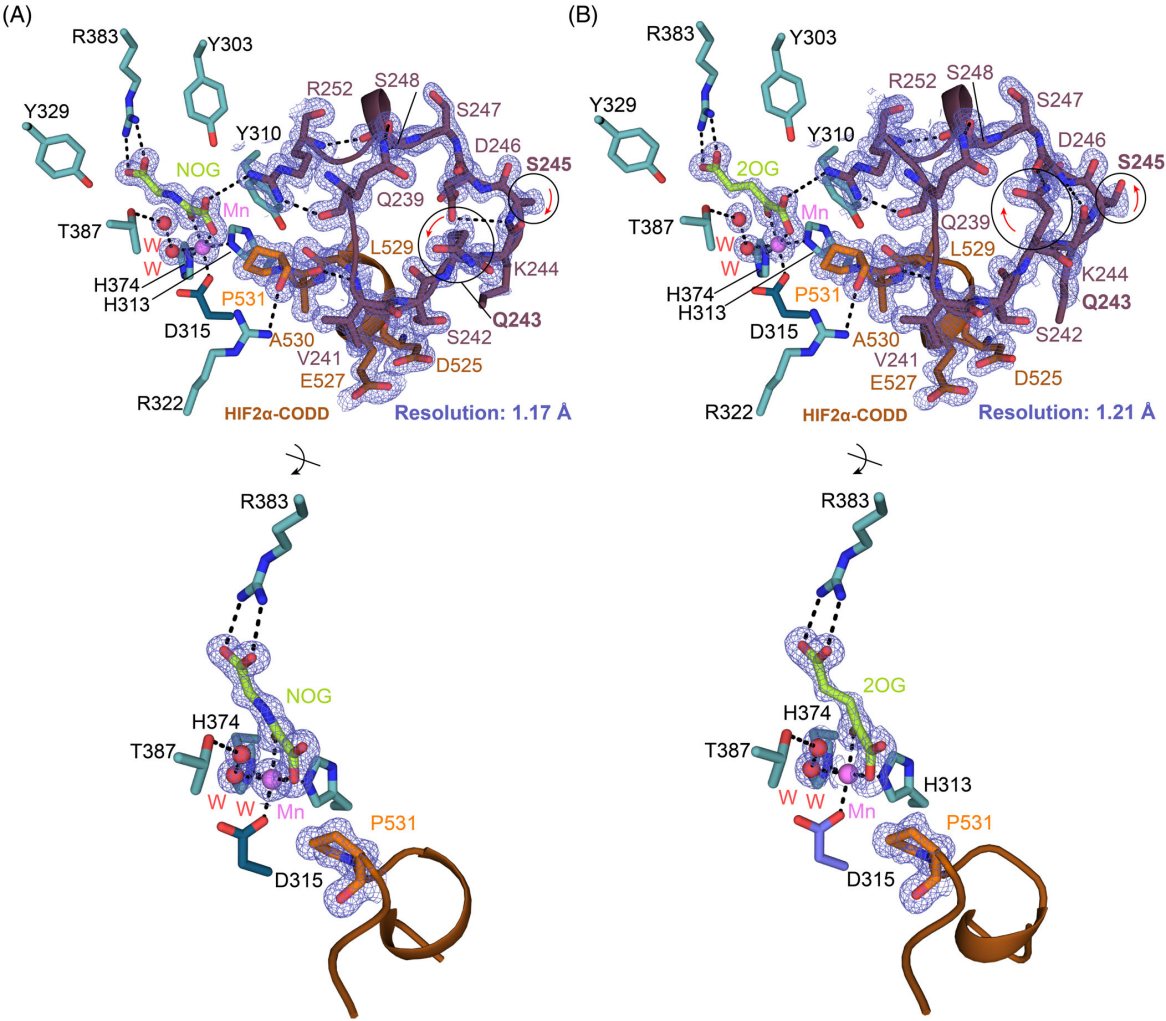
Structural basis for HIF2α‐CODD binding to PHD2. (A, B) Views from structures of PHD2_181–407_.Mn.2OG/NOG.HIF2α_523–542_‐CODD displayed as cartoons (PHD2_181–407_‐blue; HIF2α‐orange) (PDB: 7Q5V‐A and 7Q5X‐B). PHD2 residues (blue/teal), β2–β3 loop (red), 2OG/NOG (lime), and HIF2α (orange) residues are shown as sticks. The electron density map (contoured at 1.0 σ) is depicted as a mesh (blue). Key polar interactions are represented by black dashes. Waters (red) and Mn (violet) are displayed as spheres. Differences in the β2–β3 loop conformation in the two structures are highlighted by a black circle and a red arrow. Note the C4‐*endo* conformation of the substrate proline ring in both structures. CODD, C‐terminal oxygen‐dependent degradation; HIF, hypoxia‐inducible factor.

Here, we report high‐resolution crystal structures of the truncated catalytic domain of PHD2 (residues 181–407) in complex with HIF2α‐CODD (residues 523–542), a manganese ion, and 2OG or its close isostere N‐oxalylglycine (NOG) (Figures 1C and 2). The structures inform on differences in HIFα‐ODD binding that may influence the different selectivity of the PHDs.

## 2 RESULTS

### 2.1 Crystallization and structural determination of the PHD2_181–407_.HIF2α_523–542_ complex

Recently, application of the random non‐standard Peptide Integrated Discovery (RaPID) platform has enabled the identification of a cyclic peptide (3C) that binds tightly to PHD2 in a non‐substrate competing manner.^28^, ^35^ 3C binds to the N‐terminal region of the PHD2_181‐426_ catalytic domain (residues 185–214) and promotes crystallization of the PHD2_181–426_.HIF1α‐CODD complex (*P*2_1_2_1_2 space group; PDB: 6YW3).^28^ We added 3C (Figures 1B and S3B) with the aim of promoting crystallization of the PHD2_181‐407_.Mn.NOG.HIF2α‐CODD complex (Table S1). A 1 mm x 100 μm x 80 μm plate morphology crystal (*P*2_1_2_1_2 space group) was obtained within 1 week that diffracted to 1.11 Å resolution at a synchrotron source (refined to 1.17 Å resolution) (PDB: 7Q5V) (Figure 2A and Table 1). These conditions also gave crystals of the analogous PHD2_181‐407_.Mn.2OG.HIF2α‐CODD complex (*P*2_1_2_1_2 space group) that diffracted to 1.19 Å resolution (refined to 1.21 Å resolution) (PDB: 7Q5X) (Figure 2B and Table 1). Although addition of the 3C promoted crystallization of the PHD2_181‐407_.HIF2α‐CODD complexes, clear electron density for 3C was not observed in the crystal structures (Figure S3B), that is there was insufficient electron density to model in 3C as reported in the PHD2_181‐426_.HIF1α‐CODD complex structure (PDB: 6YW3).

**TABLE 1:** Data collection and crystallographic processing statistics of the PHD2_181–407._HIF2α complex structures.

	PHD2_181–407_.Mn(II).NOG.HIF2α (PDB:7Q5V)	PHD2_181–407_.Mn(II).2OG.HIF2α (PDB:7Q5X)
Beamline	Diamond Light Source‐I24	Diamond Light Source‐I24
Detector	Dectris Pilatus3 6M	Dectris Pilatus3 6M
Data processing	Xia2 DIALS	Xia2 DIALS
Wavelength (Å)	0.96861	0.97962
Resolution range (Å)	43.64–1.17 (1.21–1.17)	43.48–1.21 (1.25–1.21)
Space group	*P*2_1_2_1_2	*P*2_1_2_1_2
Unit cell (Å)	130.91 38.32 42.88	130.45 38.17 42.75
Total reflections	1 764 477 (121 954)	808 554 (71 010)
Unique reflections	73 707 (7148)	66 188 (6526)
Multiplicity	23.9 (15.4)	12.2 (10.4)
Completeness (%)	99.75 (98.07)	99.94 (99.85)
Mean I/sigma(I)	11.13 (1.20)	10.05 (1.15)
Wilson B‐factor (Å^2^)	13.75	14.13
R‐merge^a^	0.1467 (3.871)	0.1317 (4.204)
R‐meas	0.1499 (3.99)	0.1376 (4.413)
R‐pim	0.03046 (0.9422)	0.03926 (1.322)
CC1/2	0.999 (0.39)	0.999 (0.432)
CC*	1 (0.749)	1 (0.777)
Reflections used in refinement	73654 (7115)	66197 (6516)
Reflections used for R‐free	3747 (345)	3329 (334)
R‐work^b^	0.1570 (0.3095)	0.1570 (0.3118)
R‐free^b^	0.1792 (0.3188)	0.1759 (0.3075)
CC (work)	0.975 (0.722)	0.973 (0.775)
CC (free)	0.961 (0.705)	0.961 (0.805)
Number of non‐hydrogen atoms	2416	2269
Macromolecules	2105	2036
2OG/NOG	13	14
Formic acid	12	44
Cl ion	1	3
Mg ion	3	3
PEG	51	17
Glycerol	13	13
Mn ion	1	1
Solvent	277	159
Protein residues	243	240
RMS (bonds, Å)	0.008	0.011
RMS (angles, °)	1.02	1.22
Ramachandran favored (%)	97.91	97.88
Ramachandran allowed (%)	2.09	2.12
Ramachandran outliers (%)	0.00	0.00
Rotamer outliers (%)	1.37	1.43
Clashscore	5.94	4.63
Average B‐factor (Å^2^)	23.66	23.02
Macromolecules	18.5	20.92
2OG/NOG	15.65	18.676
Formic acid	56.16	39.33
Cl ion	80.79	58.14
Mg ion	45.82	46.61
PEG	52.52	49.65
Glycerol	74.27	58.14
Mn ion	9.30	9.85
Solvent	32.16	33.86
Number of TLS groups	9	10

*Note*: Single crystal diffraction data were collected from samples at 100K with conventional, rotation‐based methods. Statistics for the highest‐resolution shell are in parentheses. *R*_factor_ is equal to ∑_hkl_||Fobs(_hkl_)| − |Fcalc(_hkl_)||/ ∑_hkl_|Fobs(_hkl_)| and was calculated for the working set of reflections (*R*_work_).

^a^ R‐merge (or Rsym) is equal to ∑|I‐<I>|/∑I. R‐merge represents the data quality of merged reflection data. *I* is equal to the intensity of individual measurements and <I> is equal to the average of multiple measurements.

^b^ *R*_free_ is the *R*_factor_ for 5% of the reflections which were excluded during refinement.

**FIGURE 3:**
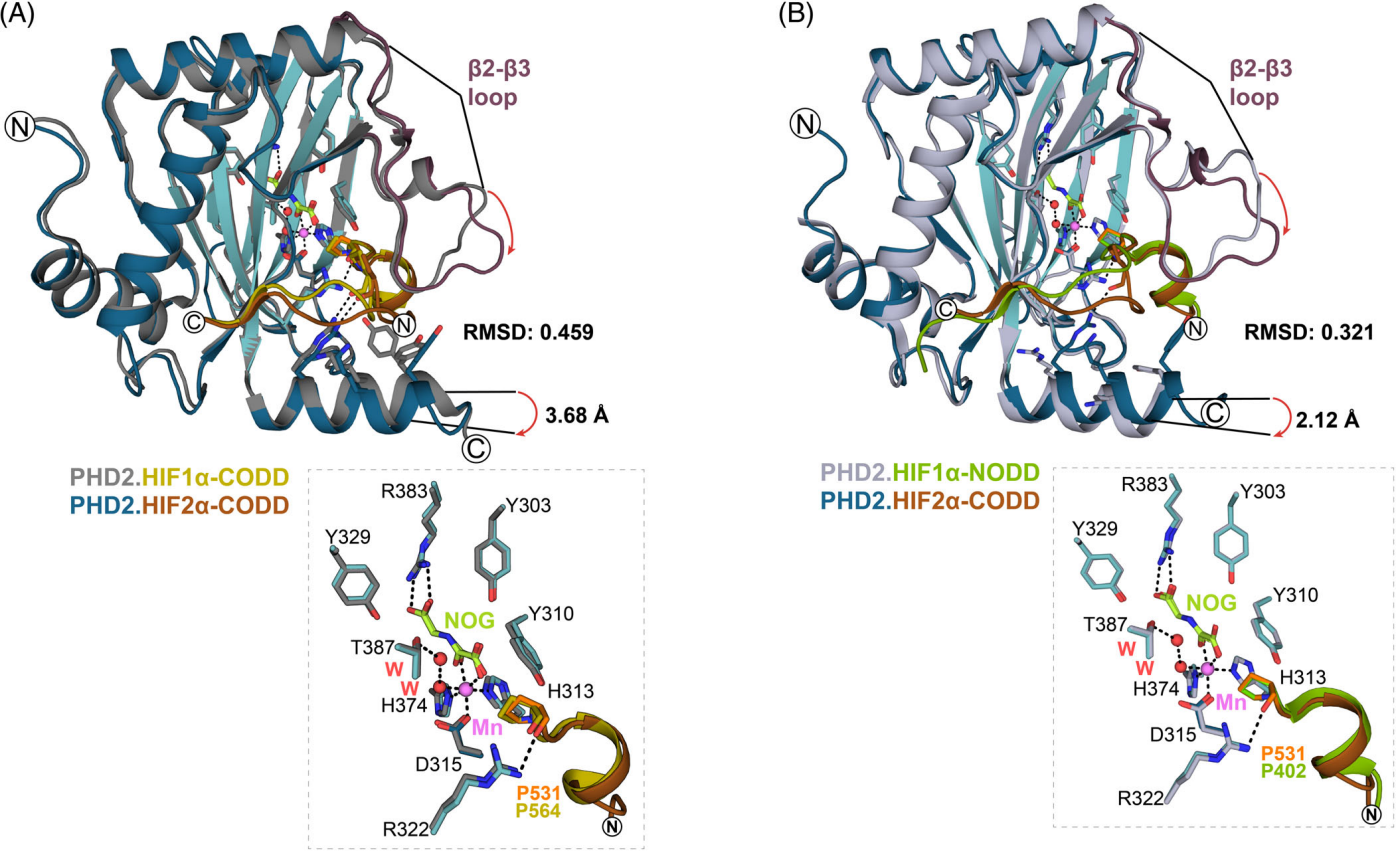
Comparison of crystal structure views of PHD2.NOG.HIF1α‐NODD/CODD complexes. (A, B) Views from structures of PHD2 (light blue‐5L9V, gray‐3HQR, and blue/cyan‐7Q5V) complexed with HIF1α_394–413_‐NODD (green‐5L9V), HIF1α_556–564_‐CODD (olive‐3HQR), and HIF2α_523–542_‐CODD (orange‐7Q5V). Polar interactions are represented by dashes (black). Waters (red) and Mn (violet) are displayed as spheres. RMSD values for aligned PHD2.HIF1α‐NODD/CODD complexes were calculated using PyMOL™. Variations in the β2–β3 loop and α4‐helix conformations are highlighted with red arrows. (A) Comparison of PHD2_181–426_.NOG.HIF1α‐CODD (3HQR) and PHD2_181–407_.NOG.HIF2α‐CODD (7Q5V) structures with a view of the active site residues and target prolines. (B) Comparison of PHD2_181–426_.NOG.HIF1α‐NODD (5L9V) and PHD2_181–407_.NOG.HIF2α‐CODD (7Q5V) structures with view of the active site residues and target prolines. CODD, C‐terminal oxygen‐dependent degradation; HIF, hypoxia‐inducible factor; NODD, N‐terminal oxygen‐dependent degradation.

Comparison of the overall PHD2_181‐426/407_.Mn.NOG/2OG.HIF2α‐CODD structures reveals conservation of the distorted double stranded β‐helix (DSBH) and associated HIFα‐ODD substrate binding elements (Figures 1B,C and 3).^11^, ^27^, ^28^, ^36^, ^37^ The NOG and 2OG structures are very similar to each other (backbone RMSD: 0.078 Å) and, to a somewhat lesser extent in terms of details, with other PHD2.HIFα‐ODD structures (Figure 3).^11^, ^27^, ^28^ In particular, variations in the conformations of α1, the β2–β3 loop, and the C‐terminal α4 regions are observed. Note that the constructs used vary in the length of their C‐terminus and in our case reversible binding of 3C may promote formation of, or stabilize, specific conformations that promote crystallization (Figure 3).^28^

The previously reported PHD2 active site chemistry is also conserved in the PHD2_181‐407_.Mn.NOG/2OG.HIF2α‐CODD structures (Figure 3),^37^ with a single manganese ion (substituting for iron) being coordinated by the side chains of His313, Asp315, and His374, as well as a well‐defined water molecule/hydroxide ion.^11^, ^27^, ^28^ The use of Mn(II) in PHD2 crystallization/inhibition is of interest given links between disease associated with Mn metabolism and erythropoiesis.^38^ 2OG and NOG bind the manganese ion in a bidentate manner via their C1 carboxylate and C2 carboxylate oxygens. The 2OG and NOG C5 carboxylates are positioned to interact with the guanidino group of Arg383 (Figures 2 and 3). The 2OG C1 carboxylate coordinates the manganese ion in the position adjacent to Pro531_HIF2α_, i.e., 2OG coordination is in an off‐line mode, suggesting that at some stage a metal‐centered rearrangement may be required to present the reactive ferryl adjacent to the oxidized Pro531_HIF2α_ C‐H bond. The pyrrolidine ring of Pro531_HIF2α_ is clearly observed in the C4‐*endo* conformation, as observed in previous PHD2‐substrate complex structures.^11^, ^27^, ^39^ However, the C4 of Pro564_HIF1α‐CODD_ is ~0.5 Å closer to the metal than the Pro531_HIF2α‐CODD,_ though whether this has any kinetic relevance is unclear (Figure 3A).^11^

Collectively, these observations reveal a conserved mode of binding for HIFα‐ODD substrate proline‐residues at the active site, including with respect to the substrate proline‐ring conformation and off‐line 2OG binding. The overall binding mode is also conserved in PHD type prolyl hydroxylases in *Trichoplax adhaerens* (*Ta*PHD),^40^ including bacteria (*Pseudomonas putida* PHD (PPHD) and *Bacillus anthracis* prolyl‐4‐hydroxylase (BaP4H)), which catalyze prolyl hydroxylation of elongation factor‐thermally unstable (EF‐Tu) (Figure S4).^41^, ^42^ This conservation is important because these features are proposed to be involved in the HIF/PHD/VHL “O_2_‐sensing” mechanism. Thus, off‐line 2OG binding may help enable the slow reaction of the PHDs with O_2_, a property proposed to be important in their “O_2_‐sensing” role.^43^ C4 proline hydroxylation is proposed to enable a stereoelectronic preference for the C4‐*exo* over the C4‐*endo* proline ring conformation, with the former being observed in PHD.HIFα‐ODD complexes and the latter in VHL.hydroxylated‐HIFα‐ODD complexes.^11^, ^39^, ^44^

Comparison of reported PHD2_181‐426_.NOG.HIF1α‐ODD complex structures (PDB: 3HQR and 5L9V) with the new PHD2_181–407_.NOG.HIF2α‐CODD (PDB: 7Q5V) structure reveals a shift in the position of the C‐terminal α4‐helix relative to the core DSBH fold, which is manifests in differences in the positions of PHD2 Thr405 and Lys402 as observed in overlaid structures. Analysis of the Thr405 Cα positions reveals a shift of ~3.7 Å in the new PHD2_181‐407_.HIF2α‐CODD complex structure (PDB: 7Q5V) compared to the PHD2_181‐426_.HIF1α‐CODD complex structure (PDB: 3HQR). Comparison of the PHD2_181‐407_.HIF2α‐CODD structure (PDB: 7Q5V) with the PHD2_181‐426_.HIF1α‐NODD structure (PDB: 5L9V) a reveals a shift of ~2.1 Å for the Lys402 Cα (Figure 3). These differences in α4 might, in part, reflect variations in the HIFα‐ODD substrate binding modes at the C‐terminal region of PHD2 and their impact on catalysis.^27^, ^29^, ^30^ However, it cannot be ruled out if these are caused by variations in crystal lattice packing, possibly relating to 3C binding.^28^

The β2–β3 loops of the PHDs are important in catalysis and in determining HIFα‐ODD substrate selectivity.^27^, ^30^, ^37^ In the absence of HIFα‐ODD substrates, the β2–β3 loop is likely conformationally mobile/disordered and principally adopts conformations that are not near the active site, including those observed by crystallography.^11^, ^27^, ^28^, ^29^, ^45^ In all reported PHD2.HIFα‐ODD structures, the β2–β3 loop folds to enclose the substrate proline residue in the active site, as is observed in our PHD2_181‐407_.HIF2α‐CODD structures (Figure 2).

Although the overall PHD2_181‐407_.HIF2α‐CODD structures with NOG and 2OG are very similar (RMSD: 0.078 Å), there are some differences in the conformations of the β2–β3 loop involving PHD2 residues Gln243‐Asp246 (Figure 2). In the 2OG.HIF2α‐CODD complex, the side chain amide NΗ_2_ group of PHD2 Gln243_β2–β3_ is positioned to form a hydrogen bond (2.74 Å) with the main chain carbonyl O atom of PHD2 Asp246 (Figure 2B). This hydrogen bond is, however, not observed in the NOG.HIF2α‐CODD complex, where Gln243_β2–β3_ is oriented away from the loop and adopts a more solvent‐exposed position (Figure 2A).

Although further work is required, given the 2OG and isostreric NOG structures have the same space group and similar crystal packing, the differences in the β2–β3 loop between them suggests that small differences at the active site region may influence the conformation of relatively distant structural elements within PHD2_181‐407_. This observation is interesting in part because recent studies on the mechanism of isopenicillin N synthase, which is structurally and mechanistically related to the 2OG‐dependent oxygenases, imply that conformational changes distant from the active site are involved in catalysis.^46^ It is also of interest because it supports the previous proposal that inhibition by 2OG mimetics involves effects on structural dynamics in addition to simple blockade of 2OG binding in the active site.^30^, ^47^, ^48^ Modeling studies on 2OG oxygenases, including demethylases, also imply the relevance of conformational changes both at and relatively distant from the active site during catalysis.^49^, ^50^ However, defining the precise effects of Fe‐binding inhibitors on the overall structural dynamics (and in some cases including complexed substrate) is technically challenging, requiring room temperature solution as well as low‐temperature biophysical crystallographic studies.^48^ Hence, in addition to studies with isolated PHDs, empirical optimization of inhibitors in a cellular context is desirable.

We compared the β2–β3 loop conformations in PHD2_181–407_.2OG/NOG.HIF2α‐CODD with those of the other PHD2.HIFα‐ODD complexes. An intra‐loop Gln243 hydrogen bond with the main chain carbonyl O of Asp246 is observed in the PHD2_181‐426_.NOG.HIF1α‐NODD (PDB: 5L9V), PHD2_181‐426_.2OG.HIF1α‐CODD (PDB: 5L9B), and our PHD2_181‐407_.2OG.HIF2α‐CODD complexes.^27^ In the case of the PHD2_181‐426_.NOG.HIF1α‐CODD (PDB: 3HQR)^11^ and PHD2_181‐407_.NOG.HIF1α‐CODD.3C (PDB: 6YW3)^28^ complexes, the side chain of Gln243 is not involved in hydrogen bonding; instead, Gln243 is in a more solvent‐exposed conformation, as observed in our PHD2.NOG.HIF2α‐CODD structure (similarly, the side chain of Ser245 adopts a different conformation in the 2OG complexed structure (PDB: 7Q5X)) when compared to the NOG complex (PDB: 7Q5V) (Figure 2). Although these crystallographic observations likely reflect snapshots of β2–β3 loop conformations in solution, they further highlight the importance of the mobility of the β2–β3 loop in catalysis.

**FIGURE 4:**
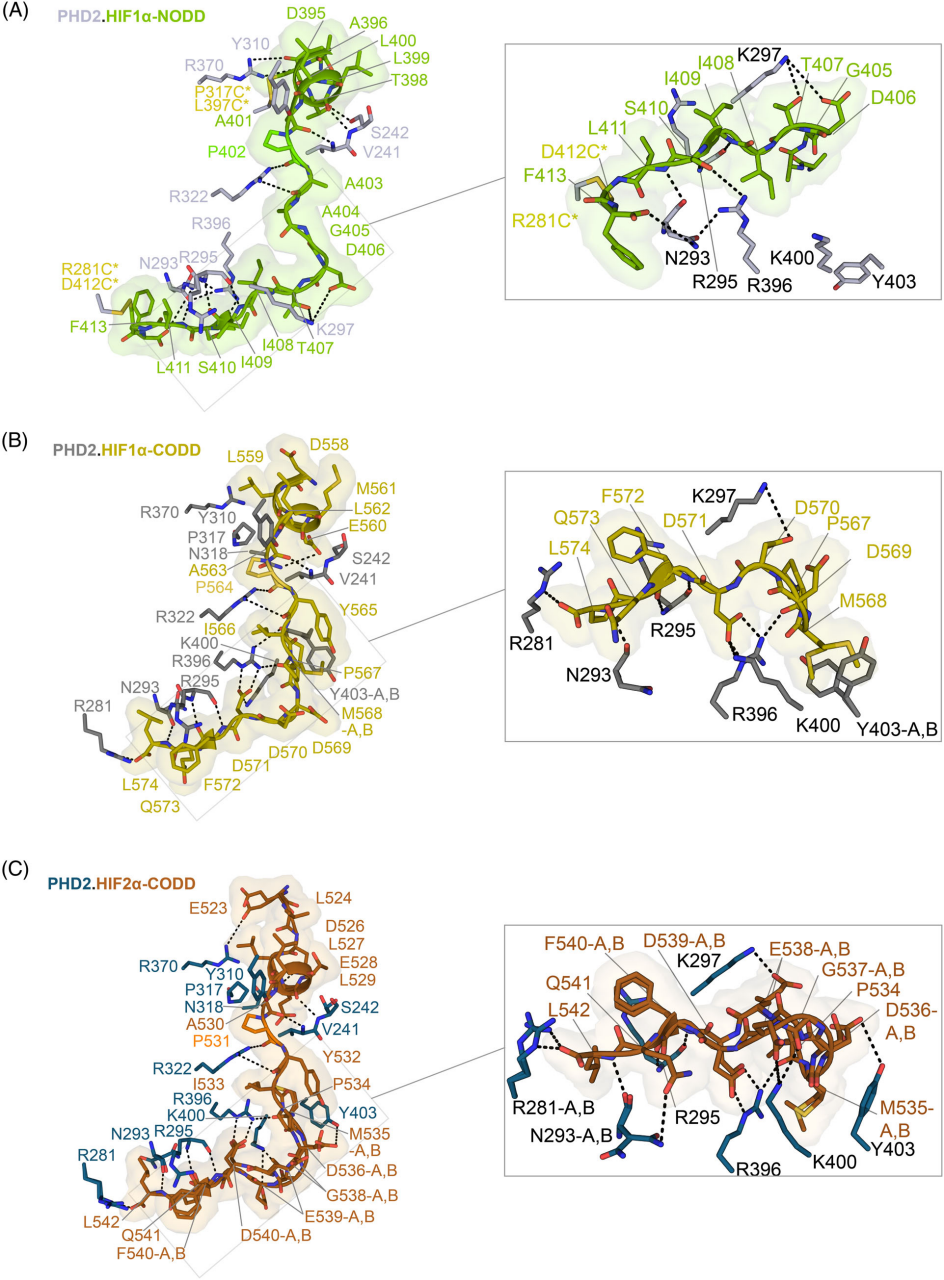
Comparison of PHD.HIFα‐ODD binding interactions. (A–C) Views showing the conformations of HIF1‐2α (HIF1α‐NODD‐green, HIF1α‐CODD‐olive, and HIF2α‐CODD‐orange) as observed by crystallography in complex with truncated PHD2 displayed as sticks with solvent‐excluded surface representation (Connolly) (PDB: 5L9V‐gray, 7Q5V‐blue, and 3HQR‐dark gray). HIF1‐2α‐ODDs are displayed as cartoons and sticks. Hydrogen bonding and electrostatic interactions are represented by black dashes. (A) Disulfide cross‐linked residues in (A), produced to enable stable complex formation, are shown as yellow sticks. CODD, C‐terminal oxygen‐dependent degradation; HIF, hypoxia‐inducible factor; NODD, N‐terminal oxygen‐dependent degradation.

Binding of the residues to the N‐terminal side of the substrate proline residue in the structures of PHD2 in complex with HIF1α_556–574_.CODD and HIF1α_394–413_.NODD involves interactions with βII, βII/III loop, β2–β3 loop, βIII, βVI–VII, and βVIII (βI–βVIII refer to the eight β‐strands of the DSBH).^27^, ^36^, ^37^ Binding of the residues to the C‐terminal side of the substrate proline residues of these peptides involves interactions with βVIII, βIII, helix α3, and the α3‐βI loop. Notably, the structures show that the CODD substrates are observed to make more polar and hydrophobic interactions with the C‐terminal α4 compared to HIF1α‐NODD (Lys400_PHD2_‐Asp571_HIF1α‐CODD_/Asp538_HIF2α‐CODD_; Tyr403_PHD2_‐Asp536_HIF2α‐CODD_; Tyr403_PHD2_‐Met568_HIF1α‐CODD_/Met535_HIF2α‐CODD_), though the mobile C‐terminal region is likely involved in catalysis in all cases.^11^, ^27^ Notably, residues Val241, Ser242, Lys244, and Ile251 of the β2–β3 loop interact with Glu560/Met561_HIF1α‐CODD_ and Thr398/Leu399_HIF1α‐NODD_, via interactions with the “XX” residues of the conserved LXXLAP motif present in all HIFα‐ODDs, highlighting the role of the β2–β3 loop in productive positioning of the different HIFα‐ODD substrates (Figure 4A,B **)**.^11^, ^27^

In our PHD2_181‐407_.HIF2α_523–542_‐CODD structures, interactions of the HIFα‐ODD residues with residues both on the N‐terminal and C‐terminal sides of the target proline peptide with PHD2 are conserved, including the interaction with α4 (Arg396_PHD2_‐Asp539_HIF2α‐CODD_) (Figure 4C). The β2–β3 loop residues (Val241, Ser242, Lys244, and Ile251) interact with Glu527/Thr528_HIF2α‐CODD_ (“XX” residues of the LXXLAP motif of HIF2α‐CODD) in a similar fashion to the previously reported PHD2 structures with HIF1α‐NODD/‐CODD.^27^

These combined observations further support a role for the β2–β3 loop in positioning the HIFα‐ODD substrates at the PHD active site, notably via interactions with the conserved LXXLAP motif in HIFα‐ODDs. Despite most of the interactions appearing to be conserved in the different HIFα‐ODDs, a striking conformational feature is observed at the C‐terminal site of HIF2α_523–542_‐CODD in both the 2OG and NOG PHD2_181‐407_.HIF2α‐CODD complex structures (PDB: 7Q5V and 7Q5X). Glu538_HIF2α‐CODD_ is observed to adopt two conformations in both structures, one of which, conformation‐A, is less solvent exposed and one of which, conformation‐B, is more solvent exposed. In conformation‐B, Glu538_HIF2α‐CODD_ projects towards a symmetry‐related chain forming a hydrogen bond with Glu538_HIF2α‐CODD_ in a symmetry‐related molecule (Figure S3A). It is possible that the conformational flexibility of Gly537_HIF2α_/Glu538_HIF2α_ unit relates to the presence of the additional Gly537 in HIF2α‐CODD on the C‐terminal side of the hydroxylated proline, compared to HIF1α/3α‐CODD and HIF1/2α‐NODD (Figure 1A). When compared with HIF1/2α‐NODD, hydrophobic Ile‐residues are in the same position as the polar Gly/Glu unit of HIF2α‐CODD; these may alter the dynamics of the enzyme‐substrate interaction (Figure 1A).^27^

**FIGURE 5:**
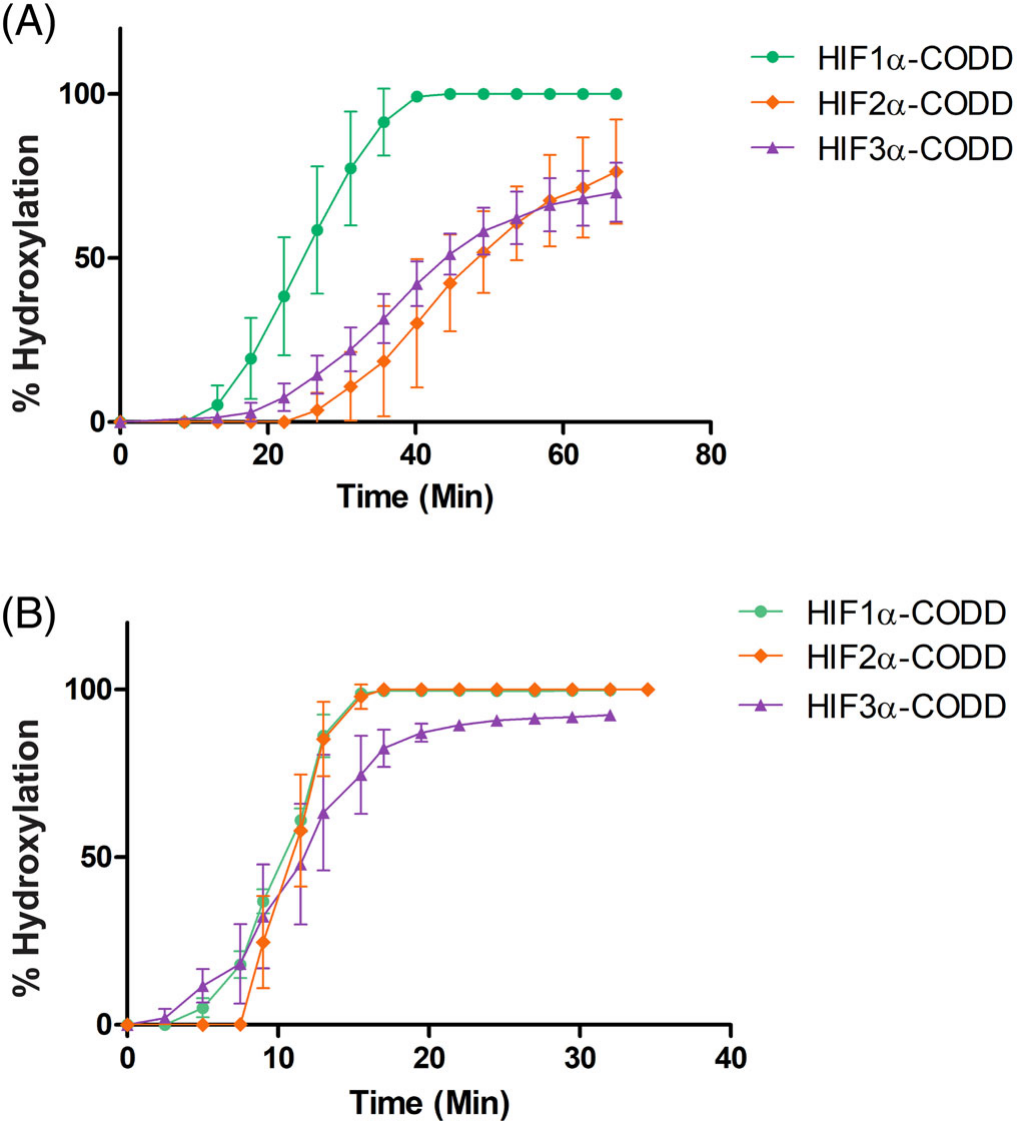
PHD2.HIFα‐CODD hydroxylation assays. (A, B) Studies on the HIFα‐CODD selectivity of PHD2_181–426_. The following CODD peptides (with C‐terminal amides) were used: HIF1α_556‐574_‐CODD (DLDLEMLAPYIPMDDDFQL), HIF2α_523–542_‐CODD (ELDLETLAPYIPMDGEDFQL) and HIF3α_484–505_‐CODD (ALDLEMLAPYISMDDDFQLN). (A) 200 μM sodium‐l‐ascorbate, 20 μM 2OG, 20 μM (NH_4_)_2_Fe(II)SO_4_, and HIF1‐3α‐CODD peptides (each at 10 μM) were mixed in a ratio of 1:1 with 300 nM PHD2_181–426_. Hydroxylation (%) was measured in real time (min) by SPE‐MS‐based enzymatic assays. (B) Single peptide control reaction conditions: 200 μM sodium‐l‐ascorbate, 20 μM 2OG, 20 μM (NH_4_)_2_Fe(II)(SO_4_), and individual HIFα‐CODD peptides (10 μM) with 300 nM of PHD2_181–426_. CODD, C‐terminal oxygen‐dependent degradation; HIF, hypoxia‐inducible factor; NODD, N‐terminal oxygen‐dependent degradation.

The above‐described differences may, at least to some extent, influence PHD2 HIFα‐isoform selectivity. To investigate the preference of PHD2 towards HIFα‐CODD substrates, we carried out assays comparing the PHD2 catalyzed hydroxylation of HIF1‐3α CODD peptides, both individually and as a mixture (Figure 5). The results with PHD2 and individual peptides showed no clear preference for the HIF1‐3α‐CODD. However, when conducting the reaction with a 1:1:1 mixture of HIF1‐3α‐CODD peptides, PHD2 showed a clear preference for HIF1α‐ over the HIF3α‐ and HIF2α‐CODDs (Figure 5A). This result supports the proposal that the presence of the additional Gly537/Glu538 unit in HIF2α‐CODD on the C‐terminal side of the hydroxylated proline, compared to HIF1α/3α‐CODD causes PHD2 to preferentially catalyze hydroxylation of HIF1α peptide over HIF2‐3α‐CODDs peptides.^51^ The Gly537/Glu538 unit in HIF2α‐CODD may also reflect differences in crystallization conditions required for the various PHD2.HIFα‐ODD complexes (Table S1).

### 2.2 Structural comparison of PHD1‐3.HIF1‐3α between crystallographic and AlphaFold predicted structures

Although structures of PHD2 in complex with HIF1‐2α‐CODD and HIF1α‐NODD are available,^11^, ^27^, ^52^ analogous structures of PHD1 and PHD3 complexed with HIFα‐ODDs are not reported. To gain insight into how structural differences between PHDs may influence isoform selectivity toward HIFα‐substrates, we built AlphaFold models^53^ of PHD1 (UniProt: Q96KS0) and PHD3 (UniProt: Q9H6Z9) and compared these with the PHD2 crystal structures (Figures S5 and S6). The structural alignments imply variations in the conformations of residues in the DSBH βII/βIII loop and β2–β3 loop regions (Figures 1B,C, S5, and S6). The predicted PHD1 DSBH βII/βIII loop region differs from that of PHD2 at two residues (Lys291_PHD2_/Val297_PHD1_ and Asn318_PHD2_/His302_PHD1_) and at four residues in the β2–β3 loop region (Ser247_PHD2_/Pro231_PHD1_, Ser248_PHD2_/Pro232_PHD1_, Asp250_PHD2_/Ser234_PHD1_, Asp246/Ile230_PHD1_). The predicted PHD3 β2–β3 loop differs at eight residues compared with PHD2 (Gln243_PHD2_/Ala66_PHD3_, Leu244_PHD2_/Arg65_PHD3_, Ser242_PHD2_/Pro64_PHD3_, Arg281_PHD2_/Leu103_PHD3_, Asn293_PHD2_/Lys115_PHD3_, Lys291_PHD2_/Tyr113_PHD3_, Gly294_PHD2_/Glu116_PHD3_, Tyr403_PHD2_/Phe225_PHD3_) (Figure S5B).

Comparison of the binding modes of HIFα‐ODDs C‐terminal to the proline substrate PHD1‐3 implies that most of the key protein:substrate interactions are conserved in the analogous PHD1 and PHD2 complexes (Figure S5). By contrast, although care should be taken not to over interpret the preliminary models, more apparent differences are observed between PHD3 and PHD2 (and by implication PHD1). In the PHD2_181‐426/407_.HIF1‐2α‐CODD structures (PDB: 3HQR and 7Q5V), Arg281_PHD2_ interacts with residues C‐terminal to the target prolines of the HIF1‐2α‐CODD substrates, that is Leu574_HIF1α_ and Leu542_HIF2α_, respectively. These interactions may not occur (or occur differently/less efficiently) in PHD3 where Arg281 is replaced by Leu103 (Figure S5D,E). Additionally, in the PHD2_181–426_.HIF1α‐NODD complex structure (PDB: 5L9V), Arg396_PHD2_ is positioned to form a polar interaction with Ser410_HIF1α‐NODD_ (Figure 5B). In the PHD3 model, Ser410_HIF1α‐NODD_ is positioned to interact with Glu116_PHD3_. Further, in the PHD3 model, the Tyr113_PHD3_ and Lys115_PHD3_ side chains clash with Phe413_HIF1α‐NODD_ (Figure S5C). The relative lack of predicted interactions between PHD3 and HIF1α‐NODD might, in part, rationalize the preference of PHD3 for HIF1‐2α‐CODD over NODD.^26^

Comparison of the HIFα‐ODD binding modes of PHD1‐3 with respect to the N‐terminal sides of the target substrate prolines (Figure S6) also implies differences in the PHD.HIFα‐ODD interactions between PHD1/PHD3 models and the PHD2 crystal structures. In PHD2_181‐426/407_.HIF1‐2α‐CODD structures (PDB: 3HQR and 7Q5V), Asn318_PHD2_ interacts with Glu560_HIF1α‐CODD_ and Asp525/Glu527_HIF2α‐CODD_; the Asn318_PHD2_ side chain is in different conformations in the HIF1α‐ and HIF2α‐CODD structures. In the predicted PHD1 model, Asn318_PHD2_ (DSBH βII/βIII loop) is replaced by His302_PHD1_ (Figure S6), which may interact differently with the HIF1‐2α‐CODDs compared to Asn318_PHD2_. In the PHD1 model with HIF1α‐NODD, Thr398_HIF1α‐NODD_ likely forms a polar interaction with His302_PHD1_; however, in the covalently cross‐linked PHD2_181‐426_.HIF‐1α‐NODD complex residues Thr398_HIF1α‐NODD_ and Asn318_PHD2_ are 5.46 Å apart, which suggests a weak interaction at this position (PDB: 5L9V). Similarly, in all reported PHD2.HIFα‐ODD complexes, Ser242_PHD2_ interacts with Leu397_HIF1α‐NODD_, Glu560_HIF1α‐CODD_, and Asp390_HIF2α‐CODD_. In PHD3, residue Ser242_PHD2_ (β2–β3 loop) is substituted by Pro64_PHD3_, the latter of which cannot make the same polar interactions (Figure S6). Pro64_PHD3_ may also alter the dynamics of the β2–β3 loop during catalysis compared with PHD1‐2.

The predicted weaker interactions of PHD3.NODD residues both on the N‐ and C‐terminal sides of the proline substrate residue may explain the low level of PHD3.HIF1‐2α‐NODD turnover observed from these substrates.^26^, ^27^ Due to the preliminary nature of the models and given the multitude of interactions in the PHD.HIFα‐ODD complexes, to what extent these structural conformational changes/induced fit processes directly influence catalysis and PHD isoform selectivity remains unclear.

## 3 DISCUSSION

Interactions between the PHDs and the HIFα‐ODDs play a central role in the hypoxic response in humans and other animals. PHD‐like prolyl‐hydroxylases are also present in certain non‐animal eukaryotes and prokaryotes, though to date these identified substrates are not HIF (like) transcription factors. In early metazoan PHD/HIF‐containing organisms, there is typically only one PHD and one HIFα, as exemplified by studies on *T. adhaerens* (Figure S4).^40^, ^54^ However, in humans and other complex HIF containing animals, there are commonly more than one PHD isoform and more than one HIFα isoform, though (typically) likely only one von Hippel–Lindau protein and one FIH.^54^ There are some subsequent bioinformatic studies that have revealed multiple PHDs and HIFα‐ODDs present in complex animals, at least in part, a reflection of the need for context‐dependent regulation of the hypoxic response.^54^, ^55^ This increased complexity may introduce vulnerabilities with respect to mutations enabling specific disease states including cancer, for example, by using modulation of one HIFα isoform in tumor progression whilst maintaining an ability to execute a robust hypoxic response, with another HIFα isoform.^31^, ^56^, ^57^ In this regard, the link between HIF2α upregulation in ccRCC (most commonly associated with VHL gene mutation) and diseases related to erythrocytosis is of interest.

Reduction of HIF2α mediated expression is the mode of action of Belzutifan which is used to treat ccRCC.^31^, ^33^ However, there is a need for new treatments for ccRCC and other diseases associated with VHL/HIFα/PHD gene mutations. Such treatments could, in principle, involve modulation of PHD.HIFα‐ODD interactions, for example, by sequestering a HIF2α‐CODD in complex with PHD, possibly in a manner that signals for a non‐VHL mediated protein degradation process, by using a small‐molecule and/or metal ion that promotes the PHD.HIF2α‐CODD interaction. The structures presented here may help in the design of such small molecules.

Since many mutations to the catalytic domain of PHD2 and HIF2α‐ODDs have been identified,^58^, ^59^, ^60^, ^61^, ^62^, ^63^, ^64^ understanding how these impact on PHD2.HIF2α‐ODD interactions is of interest in terms of understanding the molecular basis of associated diseases (Figure S2). At least some of the observed *EPAS1*/*HIF2α* mutations will likely impact on PHD catalysis as they involve residues that interact with the PHD2 active site as shown by our PHD2_181‐407_.NOG/2OG.HIF2α‐CODD structures (PDB: 7Q5V and 7Q5X) and inferred by models of PHD1‐3.HIFα‐ODD complexes (Figure S2). Strikingly, some of the clinically observed mutations (M535V, M535T, G537W, and G537R) affect the Gly537/Glu538 unit, thus likely altering PHD.HIFα‐ODD binding potentially in a manner affecting catalytic efficiency and/or HIFα‐ODD selectivity in a disease‐relevant manner. Differences in PHD1‐3.HIF1‐3α‐ODD related interactions are also important in the normal hypoxic response and knowledge of them may help enable treatments including modulation of specific sets of HIF target genes.

The combined crystallographic and NMR studies, further highlight the importance of the conformationally mobile β2–β3 loop and the C‐terminal PHD region in HIFα‐ODD hydroxylation and selectivity (Figures 2 and 4).^11^, ^27^, ^29^, ^30^, ^36^, ^45^ However, the available evidence also supports the dynamic nature of PHD.HIFα‐ODD interactions, at least in certain stages of the catalytic cycle. This means that structure‐based attempts to modulate PHD.HIFα‐ODD interactions, for example, to alter PHD isoform selectivity, should be coupled with empirical approaches in cells (note PHD.HIFα interactions likely involve other components and regions beyond the immediate PHD catalytic domain and HIFα‐ODD reactions).

The presence of an additional residue (Gly537/Glu538 unit) in HIF2α‐CODD compared to other HIFα‐ODDs, likely results in increased flexibility of HIF2α‐CODD, possibly weakening its binding to PHD2 (Figure S3A). This may, at least partially, explain the preference of PHD2 for HIF1α > HIF3α > HIF2α‐CODDs as observed in our biochemical studies (Figure 5). However, it is important to note that multiple interactions occur between the PHDs and the HIFα‐ODDs and given the dynamic nature of at least some of these interactions, it is difficult to predict the effects of individual residue changes with confidence.

By contrast, the dynamic and multivalent interaction of the PHDs with the overall HIFα‐ODDs, the chemistry in the immediate active site vicinity appears to be highly conserved in the PHDs, an observation which even extends, at least substantially, to PHD‐like enzymes with non‐HIFα substrates (Figure S4C).^41^, ^42^ The conservation includes with respect to the nature of Fe(II) and 2OG binding, including the positioning of the 2OG C1 carboxylate adjacent to the methylene of the proline‐residue that undergoes hydroxylation, an arrangement that is likely partially responsible for the unusually slow reaction of the PHDs with O_2_, though other factors also likely impact this aspect of the mechanism.^43^ Another chemically relevant conservation is the conformation of the unhydroxylated substrate proline ring at the active site, which to date has always been observed in the C4‐*endo* formation, at least in the PHD2 substrate complexes. PHD catalyzed *trans*‐4‐hydroxylation results in a bias of the proline ring to the C4‐*exo* protein conformation, due to operation of a stereoelectronic effect, as observed in pVHL‐hydroxylated‐HIFα‐ODD complex structures.^39^

Comparison of AlphaFold models of PHD1 and PHD3 with PHD2.HIF1‐2α‐CODD and PHD2.HIF1α‐NODD structures predict differences in the β2–β3 loop and C‐terminal regions apparently linked to differences in the HIFα‐ODDs binding modes, with overall fewer interactions between the HIFα‐ODDs and PHD1/3 models compared to PHD2 (Figures S5 and S6). Testing the consequences of these differences for PHD catalysis with wild‐type and clinically relevant mutated HIFα‐ODDs is the subject of ongoing work.

## 4 EXPERIMENTAL SECTION

### 4.1 Materials

Reagents, chemicals, and solvents were from Sigma‐Aldrich (Merck), Apollo Scientific, or Thermo Fisher Scientific, except where stated. The HIF1α_556–574_‐CODD (DLDLEMLAPYIPMDDDFQL), HIF2α_523–542_‐CODD (ELDLETLAPYIPMDGEDFQL), and HIF3α_484–505_‐CODD (ALDLEMLAPYISMDDDFQLN) and 3C cyclic (d‐YVWLTDTWVLSRTC)^28^, ^35^ peptides were from GL Biochem (prepared with a C‐terminal amide). Water used for cell culture was purified using a Millipore Elix® 10 system (Merck Life Sciences) and autoclave sterilized (Crystal 300‐RP25, Rodwell Engineering Group). Water used for buffers and molecular experiments was filtered purified by a 0.22‐μm Milli‐Q filtration system (Milli‐Q, Merck Life Sciences). Kanamycin (final concentration 62 mM) was prepared in water sterilized by a benchtop autoclave (LTE TouchClave II, LTE Scientific; program: 121°C and 1 Bar for 15 min) and filtered for impurities with a 0.22‐μm syringe filter (Sarstedt).

### 4.2 Expression and protein purification

The PHD2_181–407_‐pET‐28a(+) plasmid was expressed in *Escherichia coli* BL21(DE3) cells (New England Biolab Inc.).^27^ Expression was induced with 0.5 mM isopropyl β‐d‐1‐thiogalactopyranoside (IPTG) (OD_600 nm_ 0.6–1.2) at 28°C for 3–4 h.^27^ Cells were harvested and stored at −80°C until purification. Cells were freeze‐thawed at 4°C in the lysis buffer (20 mM Tris–HCl pH 7.5 RT, 0.5 M NaCl, 5 mM imidazole, and 5% (vol/vol) glycerol).^27^ DNaseI and ethylenediaminetetraacetic acid (EDTA)‐free protease inhibitor tablet (Roche) were added. Sonication (10 min total elapsed time, 3 s on/off pulse) was used for lysis (Cole‐Parmer®‐500‐Watt ultrasonic homogenizer, Cole‐Parmer). Cell lysates were then centrifuged (20,000 rpm, 4°C, JA‐25.50 rotor‐Avanti‐JHC centrifuge, Beckman Coulter); the supernatant was loaded onto a 5‐mL HisTrap™ column (GE Life Sciences) for Ni(II) affinity chromatography. A 5‐mL HisTrap™ column was charged with 5 column volumes (CVs) of 100 mM Ni(II)SO_4_, then washed with 5 CV of lysis buffer, followed by 5 CV of elution buffer (20 mM Tris–HCl pH 7.5 room temperature [RT], 0.5 M NaCl, 0.5 M imidazole, and 5% [vol/vol] glycerol), finally with 5 CV of lysis buffer.^30^ The loaded column was washed with 30 CV of wash buffer (20 mM Tris–HCl pH 7.5 RT, 0.5 M NaCl, 30 mM imidazole, and 5% [vol/vol] glycerol). Tagged PHD2_181–407_ was eluted using a step gradient (5 CV each step) of increasing elution buffer (16% [vol/vol], 34% [vol/vol], and 100% [vol/vol]).^30^ The purity of the protein fractions was analyzed by SDS‐PAGE (>90% pure material was used). His_6_‐PHD2_181–407_ fractions were concentrated to 5–6 mL volume with a concentrator (10 kDa cutoff, Amicon) at 4000 rpm and 4°C. To cleave His_6_‐tag 0.25 units of restriction grade thrombin (Novagen, Merck) and 1× thrombin cleavage buffer (10× stock of 200 mM Tris–HCl pH 8.4, 1.5 M NaCl, and 25 mM CaCl_2_, Novagen, Merck) were added to tagged PHD2_181–407_. The cleaved PHD2_181–407_ was loaded onto a Superdex® 75 gel filtration column (GE Life Sciences) pre‐equilibrated with 1 CV of 50 mM Tris–HCl pH 7.5 RT, 100 mM NaCl, and 1% (vol/vol) glycerol. Proteins were eluted with an isocratic gradient and fractions were collected and analyzed for purity by SDS‐PAGE (estimated >90%).^30^ PHD2_181–407_ was buffer exchanged with a PD‐10 desalting column (GE Life Sciences) into the final storage/crystallization buffer (50 mM Tris–HCl pH 7.5 RT, and 1% [vol/vol] glycerol).

### 4.3 PHD2.HIF2α‐CODD complex preparation

Highly purified PHD2_181–407_ via a two‐column purification strategy (affinity and SEC chromatography) was used to obtain the PHD2_181–407_.Mn(II).NOG/2OG.HIF2α_523–542_‐CODD complex crystals. Stocks of Mn(II) (100 mM), NOG pH 7–8 (80 mM), and 2OG disodium salt (100 mM) were prepared in deionized water (filter sterilized with 0.22 μm Milli‐Q filtration system, Merck Life Sciences). Cofactors/inhibitors were diluted to final concentrations of 1.2 mM‐Mn(II) and 2 mM‐NOG/2OG in the protein solution. The protein.metal.ligand mixture was pipetted directly onto lyophilized 3C cyclic peptide (weighed out at a final concentration of 2 mM into a 70‐μL final mixture volume) and left to equilibrate on a Cole‐Parmer® tube rotator (Cole‐Parmer) at 4°C for 2.5 h. PHD2_181–407_ was centrifuged at 12000 rpm (9600*g*) for 10 min at 4°C. The protein (1 mM), dissolved cofactors (1.2 mM‐Mn(II), 2 mM‐NOG/2OG), and 3C (2 mM) mixture were added to lyophilized HIF2α_523–542_‐CODD peptide (weighed out for a final concentration of 2–4 mM into a 70‐μL final mixture volume). The protein‐substrate mixtures were left overnight to equilibrate on the tube rotator at 4°C. The next day, the incubated sample was centrifuged at 14,000 rpm (18,800 *g*) and the supernatant was harvested for crystallization. The protein‐substrate sample volume was adjusted with crystallization buffer (50 mM Tris–HCl pH 7.5 RT, 1% [vol/vol] glycerol) to a final volume of 70 μL before preparation of the crystallization plates.

### 4.4 Crystallization of the PHD2_181_ _–407_.Mn(II).NOG/2OG.HIF2α_523–542_‐CODD complexes

The PHD2_181–407_.Mn(II).NOG or 2OG.HIF2α_523–542_‐CODD.3C mixtures were screened against 0.25–0.39 M magnesium formate disodium salt and 18%–22% (wt/vol) poly‐ethylene glycol (PEG) 3350 pH 7.0 (precipitant solutions were filtered; 0.22‐μm filter, Sarstedt).^28^ The protein‐substrate mixture was prepared with 1 mM PHD2_181–407_, 1 mM Mn(II)Cl_2_, 2 mM NOG/or 2OG, 2–4 mM HIF2α_523–542_‐CODD peptide, and 2 mM 3C peptide dispensed into crystallization plates (300 nL drops at 2:1 and 1:2 ratios and 200 nL drop at 1:1 ratio in Intelli‐plates, Art Robbins) with a Phoenix robot (Art Robbins) at 4°C and stored at 298K. A 1 mm × 100 μm × 80 μm (PDB: 7Q5V) and a 240 μm × 50 μm × 30 μm (PDB: 7Q5X) plate‐like crystals appeared after 1‐week of equilibration in 0.31 M magnesium formate and 16.6% (wt/vol) PEG 3350 (200 nL, 1:1 protein‐to‐well ratio, 298K). Crystals were exposed to the cryo‐protectant (reservoir solution supplemented with 20% (vol/vol) glycerol), manually looped, and cryo‐cooled into liquid‐N_2_. Crystals were stored under liquid‐N_2_ until data collection at the Diamond Light Source.

### 4.5 Solid phase extraction‐MS based enzymatic activity assays

Activity assays were conducted using a RapidFire® RF360 sampling robot (Agilent Technologies). Samples were loaded onto a C4 SPE cartridge (Agilent Technologies) and peptides were eluted with 85% (vol/vol) acetonitrile and 15% (vol/vol) water mixture added with 0.1% (v/v) formic acid. Real‐time activity assays were performed in reaction buffer containing 50 mM Tris–HCl pH 7.8 and 50 mM NaCl. Stock solutions of each component were made freshly. 100 mM stock solution of sodium‐l‐ascorbate and 50 mM stock solution of 2OG were made in water (LC–MS Grade, LiChrosolv®). 10 mM peptides stock solution were made in dimethyl sulfoxide (DMSO). To limit oxidation of Fe(II) to Fe(III), a 100 mM stock solution of (NH_4_)_2_Fe(II)(SO_4_) was made in HCl (20 mM), then diluted to 10 mM with water (LC–MS Grade, LiChrosolv®). 1 mL final volume solutions containing 200 μM sodium‐l‐ascorbate, 20 μM 2OG, 20 μM (NH_4_)_2_Fe(II)(SO_4_) and 10 μM peptide (HIF1α_556–574_‐CODD, HIF2α_523–542_‐CODD, or HIF3α_484–505_‐CODD) were prepared as control reactions. 1 mL solutions containing 200 μM sodium‐l‐ascorbate, 20 μM 2OG, 20 μM (NH_4_)_2_Fe(II)(SO_4_), and 10 μM of a 1:1:1 mixture of peptides (HIF1α_556–574_‐CODD, HIF2α_523–542_‐CODD, and HIF3α_484–505_‐CODD) were prepared for the competition reactions. About 500 μL of the substrate mixture was transferred into 96‐well polypropylene plates (Agilent Technologies). After a first injection onto the C4 SPE cartridge (Agilent Technologies), data acquisition was paused, then 500 μL of 300 nM PHD2_181–426_ in reaction buffer was added into the well to initiate the reaction. The control reactions were monitored for 32 min (one injection every 2.5 min). The competition reactions were monitored for 67 min (one injection every 5 min). The positive ion mode was used to monitor peptide charge states. RapidFire Integrator software (Agilent Technologies) was used to integrate the area of the peaks extracted from the chromatogram. Excel was used to calculate percent (%) hydroxylation of the CODD peptide substrates using the formula: % hydroxylated substrate = 100 × hydroxylated/(hydroxylated + non‐hydroxylated peptide). Oxidation of the methionine residues in the CODD sequences was 4%–6% in the no enzyme control. Every data set was normalized to a no enzyme buffer control.

### 4.6 X‐ray data analysis and software

X‐ray diffraction data were collected at Diamond Light Source synchrotron at I24 MX beamline and autoprocessed with Xia2 (DIALS, Diamond Light Source Ltd.).^65^ PHENIX.Xtriage was used to assess the data quality of the reflections.^66^ Phaser‐Molecular replacement (PHASER‐MR) was used to phase the processed diffraction data for the structures (PDB: 7Q5V and 7Q5X).^67^ A previously determined structure of PHD2 (PDB: 3HQR) was used as a search model for MR‐phasing.^11^ COOT (version 0.9.5, CCP4) was used to semi‐manual model build based on the overlaid 2mF_o_‐DF_c_ and difference mF_o_‐DF_c_ electron density maps from the phased structures (PDB: 7Q5V and 7Q5X).^68^, ^69^ The geometry of the model was adjusted based on calculated electron density maps and was improved in COOT with subsequent refinement cycles using PHENIX.Refine.^66^, ^70^ Three cycles were typically run for each refinement round before manual fitting. PHENIX.Refine was used to modify and improve the model in iterative cycles.^70^ Model improvement was assessed by the decrease in, and convergence of *R*_work_/*R*_Free_ values between cycles of refinement. MolProbity was used to assess the geometric quality of the refined model and to guide re‐building in COOT.^68^, ^71^ Resolution was defined depending on the completeness of the resolution bin (>95% in all resolution bins). PDB extract online tool (version 3.24, Research Collaboratory for Structural Bioinformatics PDB) was used to prepare coordinate and structure factors files in macromolecular CIF format (mmCIF) to be uploaded to Onedep for PDB deposition.^72^ PyMOL™ (Schrodinger) was used for graphical representation and structure alignment.

### 4.7 Quantification and statistical analysis

GraphPad prism (version 6.0) was used to plot hydroxylation over time. Multiple sequence alignments of HIF2α_523–542_‐CODD (EPAS1) sequences employed ClustalOmega (EMBL‐EBI) using the default settings. JalView (version 2.10.5) was used to generate figures.

## AUTHOR CONTRIBUTIONS

**William D. Figg, Jr.:** Conceptualization; methodology; software; data curation; investigation; validation; formal analysis; visualization; writing – original draft; writing – review and editing. **Giorgia Fiorini:** Methodology; software; data curation; investigation; validation; formal analysis; visualization; writing – original draft; writing – review and editing. **Rasheduzzaman Chowdhury:** Conceptualization; methodology; investigation; validation; writing – review and editing. **Yu Nakashima:** Software; investigation; validation; writing – review and editing. **Anthony Tumber:** Methodology; software; investigation; validation. **Michael A. McDonough:** Conceptualization; methodology; software; investigation; validation; formal analysis. **Christopher J. Schofield:** Conceptualization; methodology; investigation; validation; formal analysis; supervision; funding acquisition; visualization; project administration; resources; writing – original draft; writing – review and editing.

## CONFLICT OF INTEREST STATEMENT

The authors declare no conflicts of interest.

## Supporting information


**FIGURE S1.** Overview of the role of protein hydroxylations in the HIF‐mediated hypoxic response pathway. In the presence of sufficient O_2_, PHD1‐3, and FIH efficiently hydroxylate HIFα isoforms. PHD catalysis promotes degradation of HIFα via the ubiquitin‐proteasomal pathway in which the von Hippel–Lindau protein/elongin B/C complex plays a key role. HIF‐mediated transcription is inhibited by FIH catalysis which hinders binding of HIF to the CBP/p300 acetyltransferases. In moderate hypoxia, the PHDs are less active than FIH. In hypoxia, HIF hydroxylase activity is reduced so enabling increased levels of HIFα and formation of the transcriptionally active HIFα,β‐heterodimer.^1^, ^2^, ^3^
**FIGURE S2.** Structural locations of selected clinically observed HIF2α‐CODD variants and sequence conservation of the EPAS1 (HIF2α) C‐terminal oxygen dependent degradation domains in a set of eukaryotic organisms. (A) Predicted locations of selected predicted clinically observed HIF2α_523‐542_‐CODD variants (sticks‐yellow) on the basis of the PHD2_181–407_.Mn(II).NOG.HIF2α_523–542_‐CODD complex structure (sticks‐blue) (PDB: 7Q5V).^4^, ^5^, ^6^, ^7^, ^8^, ^9^, ^10^ PHD2 (blue) and HIF2α‐CODD (orange) are depicted as cartoons. Key polar interactions are represented by black dashes. Waters (red) and Mn (violet) are displayed as spheres. (B) Alignment of the HIF2α (EPAS1) CODD with HIF2α sequences from selected eukaryotic organisms. The percentage identities compared with the shown human HIF2α sequence are given.
**FIGURE S3.** View of the binding modes of the glycine‐glutamate unit in PHD2_181–407_.Mn.NOG.HIF2α_523–542_‐CODD and of the 3C cyclic peptide binding site. (A) Lattice packing view of the PHD2_181–407_.Mn.NOG.HIF2α_523–542_‐CODD (orange) complex crystal structure (PDB: 7Q5V). Electron density is shown as a blue mesh (contoured at 1.0 σ). Interactions of HIF2α Glu538 (conformation B) (orange) with the same residue (HIF2α Glu538, conformation B) in a symmetry‐related molecule (tan) are shown. (B) Comparison of the PHD2.Mn.NOG.HIF1α.3C (gray‐6YW3) and the PHD2_181–407_.Mn.NOG.HIF2α_523–542_‐CODD (blue/teal‐7Q5V) complex structures comparing residues that interact with the 3C cyclic peptide in the former case (density for the 3C cyclic peptide was not observed in the PHD2_181–407_.HIF2α‐CODD complex structure). Polar interactions are represented by black dashes. Waters (red) and Mn (violet) are displayed as spheres.
**FIGURE S4.** Comparison of substrate binding modes in the *Trichoplax adhaerens* PHD.*Ta*ODD, PHD2_181–407_.NOG.HIF2α‐CODD, and *Pseudomonas putida* PHD.NOG.EF‐Tu complex crystal structures. (A) Comparison structures of *Ta*PHD_21–257_.Mn(II).NOG.*Ta*ODD_477–497_ (PDB: 6F0W) and PHD2_181–407_.Mn(II).NOG.HIF2α_523–542_‐CODD (PDB: 7Q5V). Enzymes (*Ta*PHD‐green and PHD2‐blue) and substrates (*Ta*ODD‐pink and HIF2α‐orange) are shown as cartoons. The active sites, β2–β3 loops (*Ta*PHD‐green cyan and PHD2‐red), and ligands (NOG‐yellow) are displayed with key residues as sticks. Waters (red) and Mn (violet) are displayed as spheres. (B) Comparison of the *Ta*ODD_477–497_ (pink‐sticks) and HIF2α (orange‐sticks) substrate binding showing solvent‐excluded surface representation (Connolly). (A, B) Hydrogen bonding and electrostatic/polar interactions are in black dashes. (C) Two views of the off‐line NOG binding mode in the PHD2_181–407_.HIF2α‐CODD, *Ta*PHD.*Ta*ODD, and PPHD (purple).EFTu (yellow‐4IW3) complex structures. Note the conservation of the C4 *endo*‐conformation of all the substrate proline residues.
**FIGURE S5.** Comparison of binding modes of HIFα‐ODDs to PHD1‐3 involving residues to the C‐terminal side of the substrate proline residue. (A) Residues to the N‐terminal and C‐terminal sides of the substrate proline residue in HIFα‐ODD peptides are displayed on PHD1/3 model overlays. (A, B) Comparison of the conformation of the β2–β3 loop in the PHD2_181–407_.HIF2α_523‐542_ with AlphaFold (AF) models of PHD1 (cyan sticks/cartoon; UniProt: Q96KS0) and PHD3 (purple sticks/cartoon; UniProt: Q62630). (C–E) Views from crystal structures of PHD2_181–426_.HIF1α_394–413_‐NODD (PDB: 5L9V), PHD2_181–426_.HIF1α_556–574_‐CODD (PDB: 3HQR), and PHD2_181–407_.HIF2α_523–542_‐CODD (PDB: 7Q5V) displayed as cartoons and sticks (HIF1α‐NODD‐green, HIF1α‐CODD‐olive, and HIF2α‐CODD‐orange). The PHD1 (cyan; UniProt: Q96KS0) and PHD3 (purple; UniProt: Q62630) stick views are derived from AF models and are aligned with crystals for PHD2.HIFα complexes. Polar interactions in the experimentally determined PHD2 structures are represented by black dashes to enable comparison with the predicted residue conformations in the PHD1 and PHD3 models.
**FIGURE S6.** Comparison of the binding modes of HIFα‐ODDs to PHD1‐3 involving residues to the N‐terminal side of the substrate proline residue. (A–C) Views from crystal structures of cross‐linked‐PHD2_181–426_.HIF1α_394–413_‐NODD (PDB: 5L9V), PHD2_181–426_.HIF1α_556–574_‐CODD (PDB: 3HQR), and PHD2_181–407_.HIF2α_523–542_‐CODD (PDB: 7Q5V) displayed as cartoons (HIFα‐peptides) and stick views (HIF1α‐NODD‐green, HIF1α‐CODD‐olive, and HIF2α‐CODD‐orange). The PHD1 (cyan; UniProt: Q96KS0) and PHD3 (purple; UniProt: Q62630) stick views are derived from AlphaFold (AF) models aligned with crystal structures for PHD2.HIFα complexes. Polar interactions in the experimentally determined PHD2 structures are represented by black dashes to enable comparison with the predicted residue conformations in the PHD1 and PHD3 models.
**TABLE S1.** Crystallization conditions for PHD2.HIFα‐substrate complexes.

## Data Availability

Coordinates and structure factors for PHD2.HIF2α‐CODD complex structures were deposited in the RCSB Protein Data bank as: PHD2_181–407_.Mn.NOG.HIF2α_523–542_‐CODD, PDB: 7Q5V and PHD2_181–407_.Mn.2OG.HIF2α_523‐542_‐CODD, PDB: 7Q5X. AlphaFold models and HIFα sequences were accessed using UniProt: Q96KS0‐PHD1, Q9H6Z9‐PHD3, Q16665‐HIF1α, Q99814‐HIF2α, and Q9Y2N7‐HIF3α.
