# Supplementary material for: Structural basis for binding of the renal carcinoma target hypoxia‐inducible factor 2α to prolyl hydroxylase domain 2
Source: Proteins. 2023 Jul 14;91(11):1510–24. doi: 10.1002/prot.26541 (PMC10952196; doi:10.1002/prot.26541)
Supplement: Supplementary file 1 — FIGURE S1. Overview of the role of protein hydroxylations in the HIF‐mediated hypoxic response pathway. In the presence of sufficient O2, PHD1‐3, and FIH efficiently hydroxylate HIFα isoforms. PHD catalysis promotes degradation of HIFα via the ubiquitin‐proteasomal pathway in which the von Hippel–Lindau protein/elongin B/C complex plays a key role. HIF‐mediated transcription is inhibited by FIH catalysis which hinders binding of HIF to the CBP/p300 acetyltransferases. In moderate hypoxia, the PHDs are less active than FIH. In hypoxia, HIF hydroxylase activity is reduced so enabling increased levels of HIFα and formation of the transcriptionally active HIFα,β‐heterodimer. 1 , 2 , 3 FIGURE S2. Structural locations of selected clinically observed HIF2α‐CODD variants and sequence conservation of the EPAS1 (HIF2α) C‐terminal oxygen dependent degradation domains in a set of eukaryotic organisms. (A) Predicted locations of selected predicted clinically observed HIF2α523‐542‐CODD variants (sticks‐yellow) on the basis of the PHD2181–407.Mn(II).NOG.HIF2α523–542‐CODD complex structure (sticks‐blue) (PDB: 7Q5V). 4 , 5 , 6 , 7 , 8 , 9 , 10 PHD2 (blue) and HIF2α‐CODD (orange) are depicted as cartoons. Key polar interactions are represented by black dashes. Waters (red) and Mn (violet) are displayed as spheres. (B) Alignment of the HIF2α (EPAS1) CODD with HIF2α sequences from selected eukaryotic organisms. The percentage identities compared with the shown human HIF2α sequence are given. FIGURE S3. View of the binding modes of the glycine‐glutamate unit in PHD2181–407.Mn.NOG.HIF2α523–542‐CODD and of the 3C cyclic peptide binding site. (A) Lattice packing view of the PHD2181–407.Mn.NOG.HIF2α523–542‐CODD (orange) complex crystal structure (PDB: 7Q5V). Electron density is shown as a blue mesh (contoured at 1.0 σ). Interactions of HIF2α Glu538 (conformation B) (orange) with the same residue (HIF2α Glu538, conformation B) in a symmetry‐related molecule (tan) are shown. (B) Comparison of [file PROT-91-1510-s001.docx]

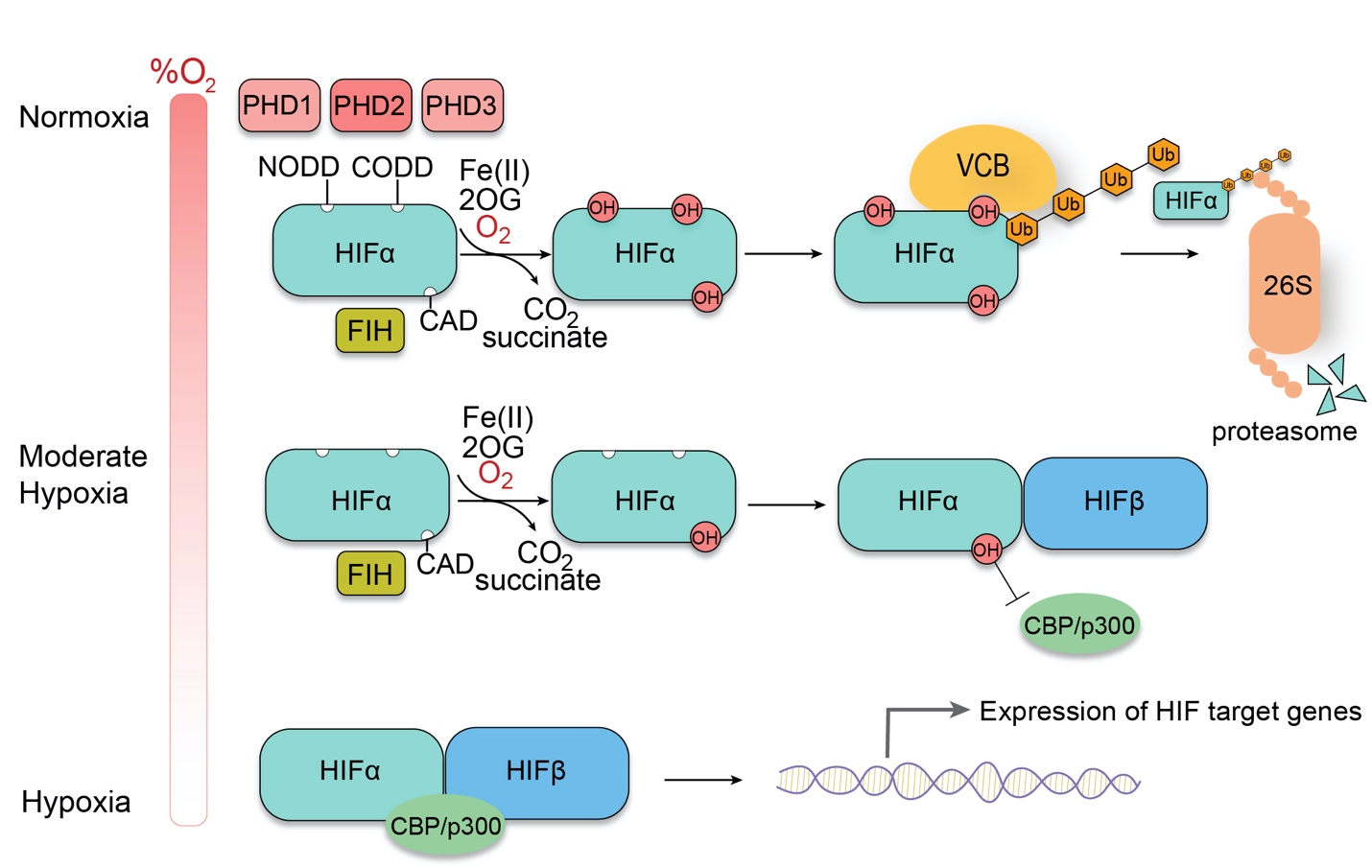


**Figure S1. Overview of the role of protein hydroxylations in the HIF-mediated hypoxic response pathway.** In the presence of O_2_, PHD1-3 and FIH efficiently hydroxylate HIFα isoforms. PHD catalysis promotes degradation of HIFα via the ubiquitin-proteasomal pathway in which the von Hippel-Lindau protein/elongin B/C complex plays a key role. HIF mediated transcription is inhibited by FIH catalysis which hinders binding of HIF to the CBP/p300 acetyltransferases. In moderate hypoxia, the PHDs are less active than FIH. In hypoxia, HIF hydroxylase activity is reduced so enabling increased levels of HIFα and formation of the transcriptionally active HIFα,β-heterodimer.^1–3^


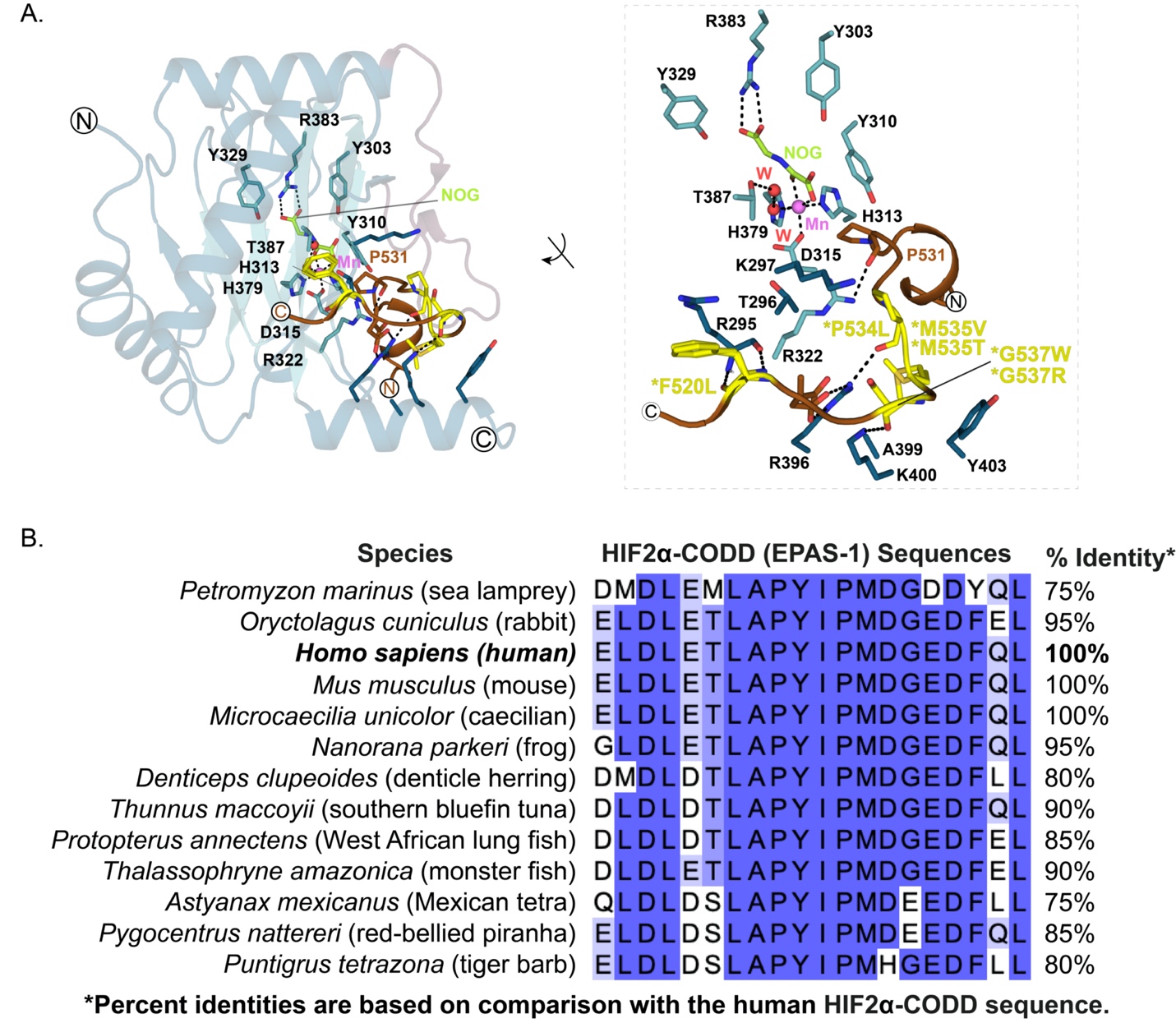


**Figure S2. Structural locations of selected clinically observed HIF2α-CODD variants and sequence conservation of the EPAS1 (HIF2α) C-terminal oxygen dependent degradation domains in a set of eukaryotic organisms.** (A) Predicted locations of selected predicted clinically observed HIF2α_523-542_-CODD variants (sticks-yellow) on the basis of the PHD2_181-407_.Mn(II).NOG.HIF2α_523-542_-CODD complex structure (sticks-blue) (PDB: 7Q5V).^4–10^ PHD2 (blue) and HIF2α-CODD (orange) are depicted as cartoons. Key polar interactions are represented by black dashes. Waters (red) and Mn (violet) are displayed as spheres. (B) Alignment of the HIF2α (EPAS1) CODD with HIF2α sequences from selected eukaryotic organisms. The percentage identities compared with the shown human HIF2α sequence are given.


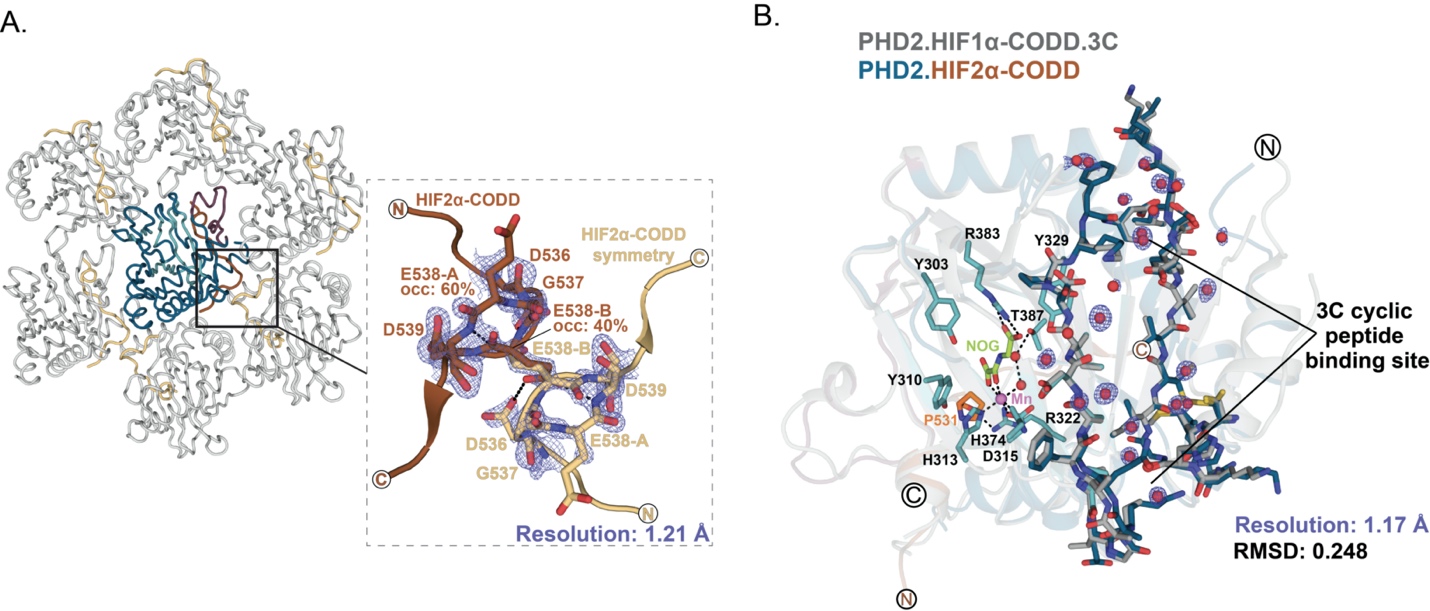


**Figure S3. View of the binding modes of the glycine-glutamate unit in PHD2_181-407_.Mn.NOG.HIF2α_523-542_-CODD and of the 3C cyclic peptide binding site**. (A) Lattice packing view of the PHD2_181-407_.Mn.NOG.HIF2α_523-542_-CODD (orange) complex crystal structure (PDB: 7Q5V). Electron density is shown as a blue mesh (contoured at 1.0 σ). Interactions of HIF2α Glu538 (conformation B) (orange) with the same residue (HIF2α Glu538, conformation B) in a symmetry-related molecule (tan) are shown. (B) Comparison of the PHD2.Mn.NOG.HIF1α.3C (grey-6YW3) and the PHD2_181-407_.Mn.NOG.HIF2α_523-542_-CODD (blue/teal-7Q5V) complex structures comparing residues that interact with the 3C cyclic peptide in the former case (density for the 3C cyclic peptide was not observed in the PHD2_181-407_.HIF2α-CODD complex structure). Polar interactions are represented by black dashes. Waters (red) and Mn (violet) are displayed as spheres.


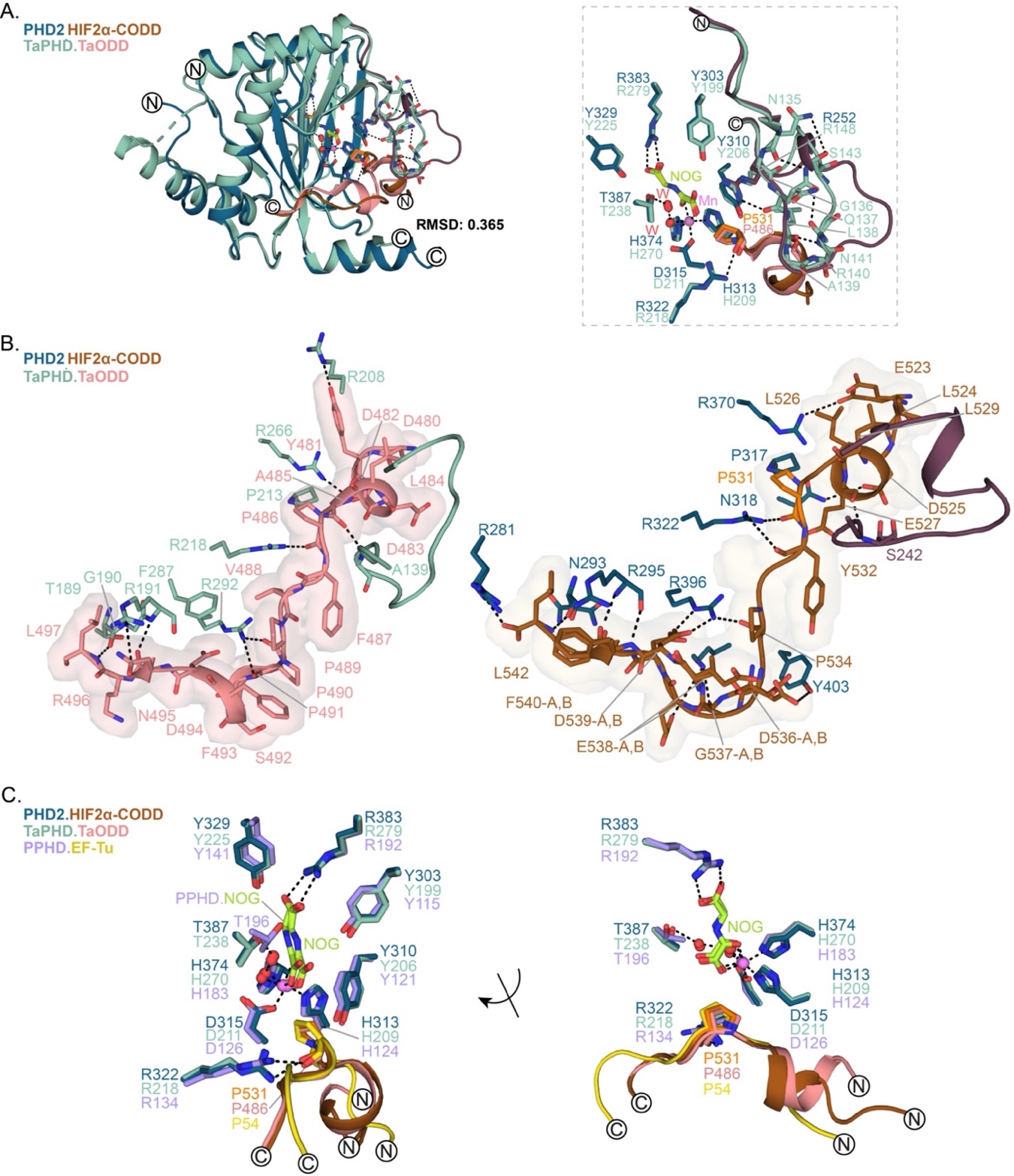


**Figure S4. Comparison of substrate binding modes in the *Trichoplax adhaerens* PHD.*Ta*ODD, PHD2_181-407_.NOG.HIF2α-CODD, and *Pseudomonas putida* PHD.NOG.EF-Tu complex crystal structures.** (A) Comparison structures of *Ta*PHD_21-257_.Mn(II).NOG.*Ta*ODD_477-497_ (PDB: 6F0W) and PHD2_181-407_.Mn(II).NOG.HIF2α_523-542_-CODD (PDB: 7Q5V). Enzymes (*Ta*PHD-green and PHD2-blue) and substrates (*Ta*ODD-pink and HIF2α-orange) are shown as cartoons. The active sites, β2-β3 loops (*Ta*PHD-green cyan and PHD2-red), and ligands (NOG-yellow) are displayed with key residues as sticks. Waters (red) and Mn (violet) are displayed as spheres. (B) Comparison of the *Ta*ODD_477-497_ (pink-sticks) and HIF2α (orange-sticks) substrate binding showing solvent-excluded surface representation (Connolly). (A-B) Hydrogen bonding and electrostatic/polar interactions are in black dashes. (C) Two views of the off-line NOG binding mode in the PHD2_181-407_.HIF2α-CODD, *Ta*PHD.*Ta*ODD, and PPHD (purple).EFTu (yellow-4IW3) complex structures. Note the conservation of the C4 *endo*-conformation of all the substrate proline residues.


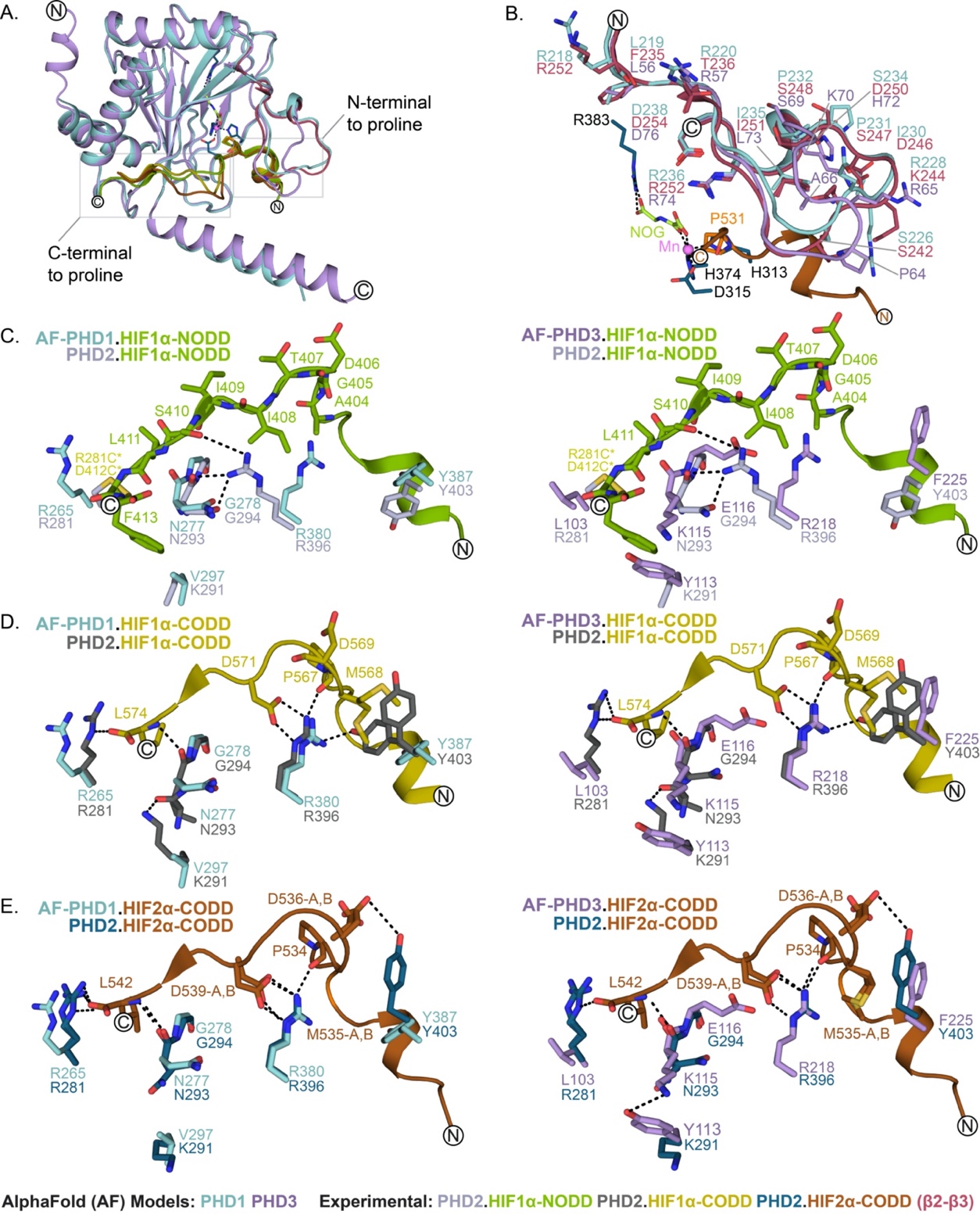


**Figure S5. Comparison of binding modes of HIFα-ODDs to PHD1-3 involving residues to the C-terminal side of the substrate proline residue.** (A) Residues to the N-terminal and C-terminal sides of the substrate proline residue in HIFα-ODD peptides are displayed on PHD1/3 model overlays. (A-B) Comparison of the conformation of the β2-β3 loop in the PHD2_181-407_.HIF2α_523-542_ with AlphaFold (AF) models of PHD1 (cyan sticks/cartoon; UniProt: Q96KS0) and PHD3 (purple sticks/cartoon; UniProt: Q62630). (C-E) Views from crystal structures of PHD2_181-426_.HIF1α_394-413_-NODD (PDB: 5L9V), PHD2_181-426_.HIF1α_556-574_-CODD (PDB: 3HQR), and PHD2_181-407_.HIF2α_523-542_-CODD (PDB: 7Q5V) displayed as cartoons and sticks (HIF1α-NODD-green, HIF1α-CODD-olive, and HIF2α-CODD-orange). The PHD1 (cyan; UniProt: Q96KS0) and PHD3 (purple; UniProt: Q62630) stick views are derived from AF models and are aligned with crystals for PHD2.HIFα complexes. Polar interactions in the experimentally determined PHD2 structures are represented by black dashes to enable comparison with the predicted residue conformations in the PHD1 and PHD3 models.

**
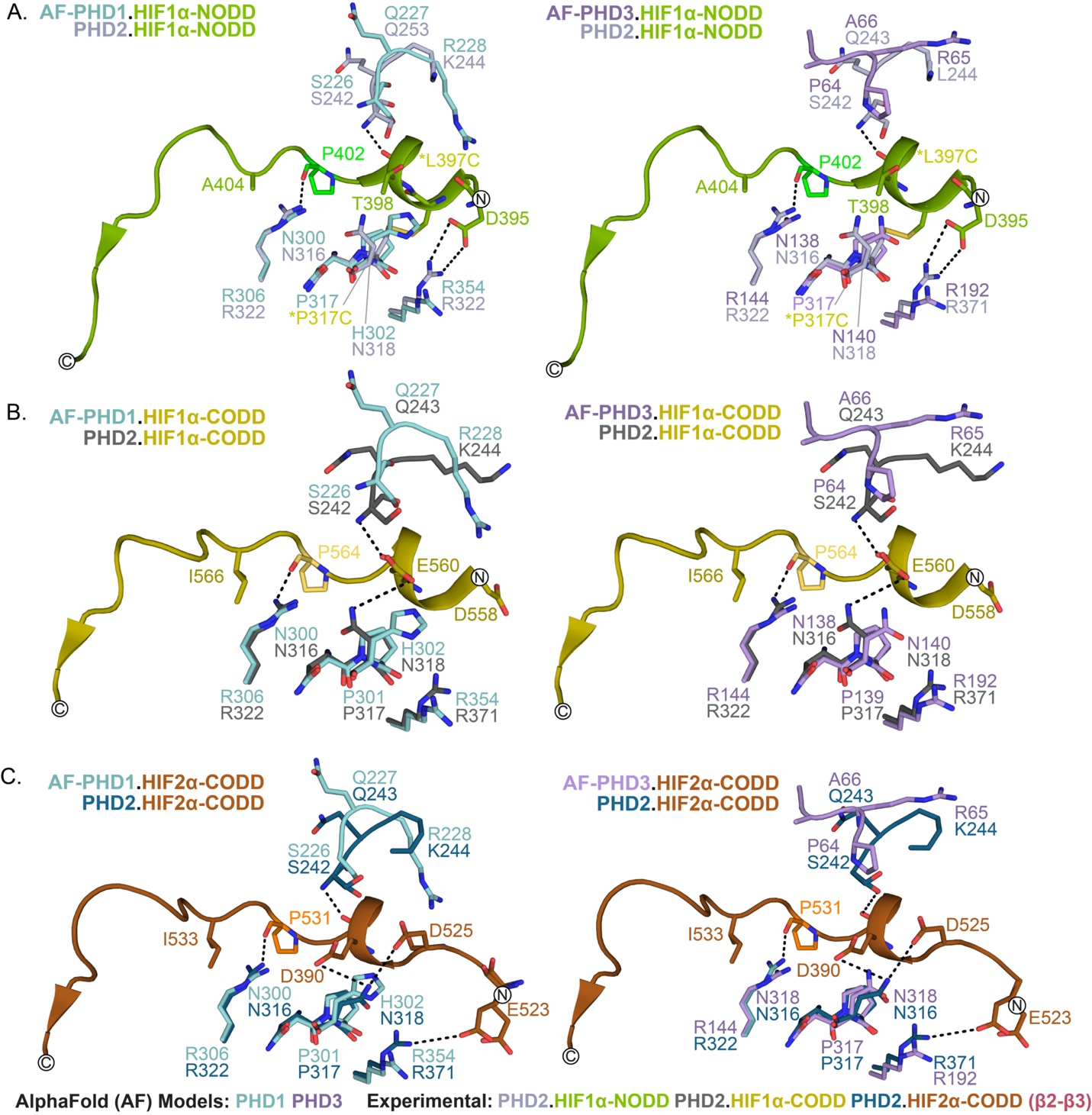
**

**Figure S6. Comparison of the binding modes of HIFα-ODDs to PHD1-3 involving residues to the N-terminal side of the substrate proline residue.** (A-C) Views from crystal structures of cross-linked-PHD2_181-426_.HIF1α_394-413_-NODD (PDB: 5L9V), PHD2_181-426_.HIF1α_556-574_-CODD (PDB: 3HQR), and PHD2_181-407_.HIF2α_523-542_-CODD (PDB: 7Q5V) displayed as cartoons (HIFα-peptides) and stick views (HIF1α-NODD-green, HIF1α-CODD-olive, and HIF2α-CODD-orange). The PHD1 (cyan; UniProt: Q96KS0) and PHD3 (purple; UniProt: Q62630) stick views are derived from AlphaFold (AF) models aligned with crystal structures for PHD2.HIFα complexes. Polar interactions in the experimentally determined PHD2 structures are represented by black dashes to enable comparison with the predicted residue conformations in the PHD1 and PHD3 models.

**Table S1. Crystallisation conditions for PHD2.HIFα-substrate complexes*.***

| **PDB** | **Protein.substrate complex** | **Crystallisation conditions** |
| --- | --- | --- |
| 3HQR | PHD2_181-426_.Mn(II).NOG.HIF1α_556-574_-CODD  *P*2_1_2_1_2 | 20% (w/v) PEG 3350, 0.2 M MgCl_2_, pH 7.5,  hanging-drop, 298K |
| 5L9B | PHD2_181-426_.Mn(II).2OG.HIF1α_556-574_-CODD  *P*2_1_ | 25% (w/v) PEG 3350, 0.2 M MgCl_2_ • 6H_2_O, 0.1 M Bis-Tris pH 5.5, sitting-drop, 293K |
| 5L9V | PHD2_181-426_.Mn(II).NOG.HIF1α_394-413_-NODD  *P*2_1_ | 20% (w/v) PEG 3350, 0.2 M NH_4_Cl, pH 7.5,  sitting-drop, 293K |
| 5LA9 | PHD2_181-426_.Mn(II).NOG.HIF1α_394-413_-NODD  *P*2_1_2_1_2 | 2.0 M (NH_4_)_2_SO_4_, 0.1 M HEPES pH 7.5,  sitting-drop, 293K |
| 5LAS | PHD2_181-426_.Mn(II).NOG.HIF1α_394-413_-NODD  *P*21 | 20% (w/v) PEG 6000, 0.1 M citrate pH 5.0,  sitting-drop, 293K |
| 6YW3 | PHD2_181-426_.Mn(II).NOG.HIF1α_556-574_-CODD.3C  *P*2_1_2_1_2 | 19-23% (w/v) PEG 3350, 0.25 M Li_2_SO_4_, 2 mM MnCl_2_, pH 6.5  sitting-drop, 293K |
| 7Q5V | PHD2_181-426_.Mn(II).NOG.HIF1α_556-574_-CODD  *P*2_1_2_1_2_1_ | 18-22% (w/v) PEG 3350, 0.25-0.34 M MgHCO_2_^-^, pH 7.0,  sitting-drop, 298K |
| 7Q5X | PHD2_181-426_.Mn(II).2OG.HIF1α_556-574_-CODD  *P*2_1_2_1_2_1_ | 18-22% (w/v) PEG 3350, 0.25-0.34 M MgHCO_2_^-^, pH 7.0,  sitting-drop, 298K |

**References**

1. Schofield CJ, Ratcliffe PJ. Oxygen sensing by HIF hydroxylases. *Nat Rev Mol Cell Biol*. 2004;5(5):343-354.

2. Kaelin WG, Ratcliffe PJ. Oxygen Sensing by Metazoans: The Central Role of the HIF Hydroxylase Pathway. *Mol Cell*. 2008;30(4):393-402.

3. Semenza GL. Hydroxylation of HIF-1: Oxygen sensing at the molecular level. *Physiology*. 2004;19(4):176-182.

4. Furlow PW, Percy MJ, Sutherland S, et al. Erythrocytosis-associated HIF-2α Mutations Demonstrate a Critical Role for Residues C-terminal to the Hydroxylacceptor Proline. *J Biol Chem*. 2009;284(14):9050-9058.

5. Gale DP, Harten SK, Reid CDL, Tuddenham EGD, Maxwell PH. Autosomal dominant erythrocytosis and pulmonary arterial hypertension associated with an activating HIF2α mutation. *Blood*. 2008;112(3):919-921.

6. Maurizio Martini, Luciana Teofili, Tonia Cenci, et al. A novel heterozygous HIF2AM535I mutation reinforces the role of oxygen sensing pathway disturbances in the pathogenesis of familial erythrocytosis. *Haematologica*. 2008;93(7 SE-Brief Reports):1068-1071.

7. Percy MJ, Beer PA, Campbell G, et al. Novel exon 12 mutations in the HIF2A gene associated with erythrocytosis. *Blood*. 2008;111(11):5400-5402.

8. Percy MJ, Furlow PW, Lucas GS, et al. A Gain-of-Function Mutation in the HIF2A Gene in Familial Erythrocytosis. *N Engl J of Med*. 2008;358(2):162-168.

9. Perrotta S, della Regione F. The HIF2A Gene in Familial Erythrocytosis. *N Engl J Med*. 2008;358(18):1965-1967.

10. Richard van Wijk, Scott Sutherland, Annet C.W. Van Wesel, et al. Erythrocytosis associated with a novel missense mutation in the HIF2A gene. *Haematologica*. 2010;95:829-832.
